# Machine learning-driving optimization and spatial assembly of a cell-free system for high-yield liquiritigenin production

**DOI:** 10.1007/s44307-026-00103-0

**Published:** 2026-03-27

**Authors:** Fei Liu, Si-Bo Zhao, Yan-Hua Liu, Jun-Feng Li, Nuo-Qiao Lin, Meihereayi Mutailifu, Pei Xu, Jian-Zhong Liu

**Affiliations:** State Key Laboratory of Biocontrol, School of Life Sciences, Sun Yat-Sen University, Guangzhou, 510275 People’s Republic of China

**Keywords:** Liquiritigenin, CFME, CFPS-ME, Machine learning, Optimized conditions, Enzyme assembly

## Abstract

Liquiritigenin is a medicinal flavonoid whose production is constrained by inefficient plant extraction and complex chemical synthesis. To overcome this, we developed a modular cell-free multi-enzyme system for its efficient biosynthesis from tyrosine, integrating spatial enzyme assembly with machine learning-guided optimization. Using a combined cell-free metabolic engineering (CFME) and cell-free protein synthesis-driven metabolic engineering (CFPS-ME) approach, we screened and optimized five key pathway enzymes to establish a one-pot reaction. The optimal enzyme combination (phenylalanine ammonia-lyase from *Zea mays*, 4-coumarate-coenzyme A ligase 4 from *Arabidopsis thaliana*, chalcone synthase from *Glycine max*, chalcone reductase from *Medicago sativa*, chalcone flavonone isomerase from *Zea mays*) was identified through systematic screening and ratio optimization. After Plackett–Burman and steepest-ascent experiments, three rounds of iterative machine learning fine-tuned key parameters, including enzyme ratios and cofactor concentrations, yielding 155.32 ± 14.39 mg/L. Spatial enzyme assembly was further enhanced via covalent peptide tags and scaffold proteins (γPFD-SpyCatcher) under CFME. Combining CFPS-ME with scaffold-assisted co-immobilization significantly boosted production, reaching a final titer of 439.42 ± 19.53 mg/L. This study demonstrates that machine learning-driven optimization and spatial assembly of multienzyme complexes is a powerful approach for cell-free biosynthesis.

**Supplementary Information:**

The online version contains supplementary material available at 10.1007/s44307-026-00103-0.

## Introduction

Liquiritigenin is a naturally occurring flavonoid derived from the rhizomes of *Glycyrrhiza* species (*Fabaceae*) (Sayre et al. 2016). It exhibits a broad spectrum of biological activities, including cardioprotective (Khavkin et al. 2024), antidiabetic (Shamsudin et al. 2022; Zhu et al. 2018), antioxidant (Fatoki et al. 2023), anti-inflammatory (Liu et al. 2025; Leite et al. 2022), and antitumor effects (Liu et al. 2023; Meng and Lin 2019; Deng et al. 2023). Within the plant polyphenol biosynthetic network, liquiritigenin serves as a central intermediate branching into flavonol and isoflavone pathways and functions as an essential precursor for diverse flavonoid derivatives (Rodriguez et al. 2017). Due to its pharmacological potential and versatile bioactivities, liquiritigenin has garnered considerable attention in the fields of food, pharmaceuticals, and synthetic biology. Currently, its production relies on plant extraction and chemical synthesis, which are hampered by limited raw material availability, low yields, and complex purification processes (Yin et al. 2020). Recent studies have reported the microbial synthesis of liquiritigenin. Researchers successfully constructed an engineered Saccharomyces cerevisiae strain that produces liquiritigenin by introducing the chalcone reductase (MsCHR) gene from *Medicago sativa* and other pathway enzyme genes. When 1.5 mmol/L *p*-coumaric acid was supplemented, the strain yielded 13.5 mg/L of liquiritigenin(Yan et al. 2007). Furthermore, using *p*-coumaric acid-high producing engineered *S. cerevisiae* as the chassis, researchers engineered a strain capable of de novo liquiritigenin synthesis via a multi-pathway enzyme-screening strategy, yielding 5.3 mg/L (Rodriguez et al. 2017). In contrast, the improvement of liquiritigenin titer has been realized through multiple strategies. For example, the combination of enzyme cocktail optimization and PDZ-PDZlig (protein-peptide) self-assembly-mediated chalcone synthase-chalcone reductase (CHS-CHR) coupling strategy successfully increased the liquiritigenin yield to 44.5 mg/L in *Escherichia coli.* (Li et al. 2021). Using *Yarrowia lipolytica* with optimized metabolic flux as the chassis, a yield of 62.4 mg/L was achieved in a fermenter via the optimization of multi-gene combination and regulatory strategies (Akram et al. 2021). Furthermore, Deng et al. (2025) recently engineered the galactose-inducible system of *S. cerevisiae* and enhanced dual nicotinamide adenine dinucleotide phosphate (NADPH) supply, significantly improving its biosynthetic efficiency. However, the production efficiency of liquiritigenin remains lower compared to that of other flavonoids, indicating that it warrants further investigation.

The emergence of cell-free biosynthesis (CFBS) has opened new opportunities to address the limitations of living cell systems, such as limited metabolic flux and slow catalytic rates (Ullah et al. 2023; Bundy et al. 2018). Cell-free systems also avoid the lengthy and expensive enzyme purification process needed for in vitro catalysis, thereby improving efficiency and scalability (Zhang et al. 2023; Lee and Kim 2024). As a result, cell-free metabolic engineering (CFME) has been successfully used to produce high-value compounds like cinnamyl alcohol (Zhang et al. 2023), styrene (Grubbe et al. 2020), limonene (Dudley et al. 2020), pinene (Niu et al. 2020), n-butanol (Karim and Jewett 2016) and ribosomally synthesized and post-translationally modified peptides (RiPPs) (Liu et al. 2024a, b). However, low enzyme concentrations (around 10 mg/mL) in CFME limit the industrial use of crude extract-based CFME. Our previous study developed a new cell-free biosynthesis system that combines chassis cell extracts with purified Spy-cyclized enzymes to enhance local enzyme concentration and increase CFME yields (Niu et al. 2021).

In nature, metabolic pathways are often organized into multienzyme complexes and are incorporated into the crowded macromolecular environment, resulting in higher local enzyme concentration. Inspired by the natural system, compartmentalization and scaffolding have been successfully applied in a multi-enzyme cascade reaction based on purified enzymes (Ledesma-Fernandez et al. 2023; Velasco‐Lozano et al. 2020; Zhang et al. 2018; Zhao et al. 2021; Liu et al. 2024a, b; Wang et al. 2024; Peng et al. 2021). However, these co-localization strategies have not been used in the crude extract-based CFME.

Recently, machine learning (ML), one of the branches of artificial intelligence, has gained great interest in the field of biotechnology and biomanufacturing. It has been considered as a powerful tool for process optimization and control strategies (Wang et al. 2025). An artificial intelligence system was developed to automatically regulate the feeding. In metabolic engineering, machine learning (ML) can decipher the complex interactions between metabolic modules, thereby providing precise guidance for gene integration strategies to achieve substantial improvements in product yield(Tu et al. 2026). Similarly, the integration of machine learning with cell-free synthetic biology systems is advancing enzyme engineering into a new phase of rational design. Such platforms enable efficient exploration of protein sequence space and guide the directed evolution of enzymes(Landwehr et al. 2025). Additionally, combining droplet microfluidic screening with ML predictive models allows for the streamlined optimization of cell-free systems, drastically cutting costs while boosting protein expression yields(Zhu et al. 2025).

**Fig. 1:**
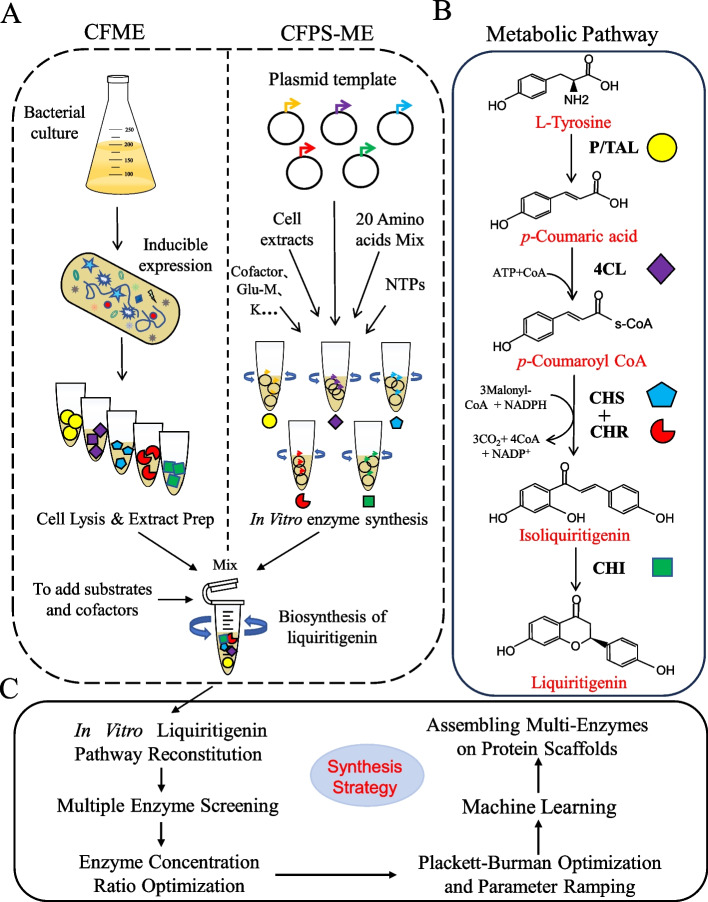
Scheme of the cell-free biosynthetic pathway for liquiritigenin. **A** Schematic diagram of the two biosynthetic approaches, cell-free metabolic engineering (CFME) and cell-free protein synthesis-driven metabolic engineering (CFPS-ME). Strategies such as multi-enzyme screening, optimization of enzyme ratios, and cofactor balancing were employed to enhance liquiritigenin production. **B** The five-enzyme cascade reaction from L-tyrosine to liquiritigenin. P/TAL, tyrosine ammonia-lyase; 4CL, 4-coumarate-CoA ligase; CHS, chalcone synthase; CHI, chalcone isomerase; CHR, chalcone reductase. **C** Stepwise enhancement of cell-free biosynthetic liquiritigenin production using a combinatorial strategy

In this study, we reconstructed and activated the liquiritigenin biosynthetic pathway using cell-free metabolic engineering (CFME) and cell-free protein synthesis-driven metabolic engineering (CFPS-ME) (Fig. 1). ML-guided optimization of the CFB process was conducted. Finally, the introduction of a protein scaffold into the crude extract-based CFPS-ME system resulted in a dramatic enhancement of liquiritigenin production.

## Materials and methods

### Bacterial strains and plasmids

All bacterial strains and plasmids used in this study are listed in Table S1. The primer sequences are given in Table S2. *Escherichia coli* BL21 Star (DE3) was used as the host strain for the expression of the pathway enzymes.

All genes were synthesized after codon optimization for *E. coli* by Tsingke Biotechnology Co., Ltd. (Beijing, China) and cloned into pET32a. These genes were listed in Table 1.

**Table 1:** The information of pathway genes used in this study

Gene	Source	GenBank	Size
Phenylalanine ammonia-lyase (ZmPAL)	*Zea mays*	AAL40137.1	2112
4-Coumarate-coenzyme A ligase2 (Pc4CL2)	*Petroselinum crispum*	CAA31697.1	1635
Chalcone reductase (MsCHR)	*Medicago sativa*	AAB41555.1	939
Chalcone reductase (PhCHSA)	*Petunia hybrida*	CAA32731.1	1170
Chalcone flavonone isomerase (ZmCHI)	*Zea mays*	CAA80441.1	696
4-Coumarate-coenzyme A ligase1 (At4CL1)	*Arabidopsis thaliana*	AF106084.1	1683
4-Coumarate-coenzyme A ligase (At4CL2)	*Arabidopsis thaliana*	AAD47192.1	1491
4-Coumarate-coenzyme A ligase 4 (At4CL4)	*Arabidopsis thaliana*	AAM19949	1479
4-Coumarate-coenzyme A ligase 3 (Os4CL3)	*Oryza sativa*	NP_001396278.1	1677
Tyrosine ammonia lyase (RgTAL)	*Rhodotorula glutinis*	KF765779.1	2082
Tyrosine ammonia lyase (FjTAL)	*Flavobacterium johnsoniae*	SCV44818.1	1521
Chalcone synthase (EbCHS)	*Erigeron breviscapus*	AED02599.1	1197
Chalcone synthase (GmCHS)	*Glycine max*	AAA33950.1	1170
Chalcone isomerase (MsCHI)	*Medicago sativa*	AAB41524.1	669
Tyrosine ammonia lyase (RgTAL^214c^)	*Rhodotorula glutinis*	KF765779.1	2082

### Preparation of cell extracts

Cell extracts were prepared according to the protocol (Zhuang et al. 2020) with slight modifications. Briefly, *E. coli* BL21 Star (DE3) cells without pathway enzymes were cultured in 1 L 2 × YTPG medium at 37 °C. Strains harboring plasmids for pathway enzyme expression were grown in ZYM-5052 auto-induction medium (1% Tryptone, 0.5% yeast extract, 25 mM Na_2_HPO_4_, 25 mM KH_2_PO_4_, 50 mM NH_4_Cl, 5 mM Na_2_SO_4_, 0.5% glycerol, 0.05% glucose, 0.2% L-arabinose, 2 mM MgSO_4_, 0.2 × trace metal solution. 1000 × trace metal solution contains 50 mM FeCl_3_, 20 mM CaCl_2_, 10 mM each of MnCl_2_ and ZnSO_4_, and 2 mM each of CoCl_2_, CuCl_2_, NiCl_2_, Na_2_MoO_4_, Na_2_SeO_3_, and H_3_BO_3_ in 60 mM HCl). Cultures (50 mL) were inoculated in 250 mL flasks and incubated at 37 °C with shaking at 250 rpm. Cell density was monitored by UV spectrophotometry. When OD600 reached 0.6–0.8, 0.3 mM IPTG was added to induce protein expression, followed by overnight incubation at 16 °C. Cells were harvested by centrifugation (5500 g, 15 min, 4 °C), washed three times with prechilled S30 buffer (10 mM Tris–acetate, pH 8.2; 14 mM magnesium acetate; 60 mM potassium glutamate), and stored at −80 °C after snap-freezing in liquid nitrogen. For extract preparation, thawed cells were resuspended in 1 mL of S30 buffer per gram of wet biomass. The suspension was disrupted on ice using sonication (220 W, 3 s on/5 s off, 10 min total). Lysates were clarified by centrifugation (18,000 g, 15 min, 4 °C), and the supernatant was aliquoted, snap-frozen, and stored at −80 °C until use. Protein concentration was determined using the Bradford assay.

### Cell-free protein synthesis (CFPS)

CFPS was performed according to a published protocol (Oza et al. 2019) with minor modifications. The reaction was carried out in a 57 mM HEPES buffer system containing 16 ng/μL template DNA, 10 mg/mL *E. coli* extract, 8 mM magnesium glutamate, 10 mM ammonium glutamate, 130 mM potassium glutamate, 1.2 mM ATP, 0.85 mM GTP/UTP/CTP, 0.034 mg/mL folinic acid, 0.171 mg/mL tRNA, 33.33 mM phosphoenolpyruvate, 2 mM of each of the 20 standard amino acids, 0.40 mM NAD, 0.27 mM CoA, 1 mM putrescine, and 1.5 mM spermidine. To further enhance protein yield, 60 mM maltodextrin, 30 mM D-ribose, and 3.5% (w/v) PEG8000 were added (Garenne et al. 2021). After incubation for 5–8 h at 30℃, the reaction solution was centrifuged at low speed (4 °C) and the supernatant was collected. Total protein concentration of CFPS-derived enzymes was determined using the Bradford assay.

### Cell-free biosynthesis of liquiritigenin

The cell-free biosynthesis reaction (Velasco‐Lozano et al. 2020; Dudley et al. 2019) was carried out in a mixture containing 8 mM magnesium glutamate, 10 mM ammonium glutamate, 134 mM potassium glutamate, S30 buffer (10 mM Tris, 14 mM magnesium acetate, 60 mM potassium acetate, pH 8.0), 1.5 mM ATP, 1.5 mM CoA, 1.5 mM NADPH, 10 mM K_2_HPO_4_, and the different concentrations of the pathway enzymes. For CFPS, the pathway enzymes were obtained from the CFPS reaction. For CFME, the pathway enzymes were obtained from the cell extract. The substrate L-tyrosine (2 mM) was added to initiate the reaction, which was incubated at 37 °C for 24 h.

### Plackett–Burman experimental design

The Plackett–Burman (PB) experimental design is a two-level multifactor screening method that does not take into account the interactions among different factors. It is suitable for rapidly and effectively screening out several important factors from numerous investigated variables for further study.

The influence of each factor on the target variable *y* is described by the following multiple linear equation:

1 $$y\;=\;\beta\;+\;E_{X1}X_1\;+\;E_{X2}X_2\;+\;E_{X3}X_3\;+\;\cdots\;+\;E_{xi}X_i$$

2 $$E_{xi}\;=\;\left(\sum M_{i+}-\sum M_{i-}\right)/N$$

where *y* represents the target experimental value, *β* is the intercept, *E*_*Xi*_ is the regression coefficient, and *Xi* denotes the investigated factors. In the Plackett–Burman design, each factor is set at two levels, high and low, represented by + 1 and − 1, respectively. *M*_*i*+_ represents the target experimental value of factor *X*_*i*_ at the high level (+ 1), *M*_*i-*_ represents the target experimental value at the low level (− 1), and N represents the total number of experiments. In this study, the factors affecting the cell-free de novo biosynthesis of liquiritigenin are shown in Table 2.

**Table 2:** Plackett–Burman experimental design for liquiritigenin production

Variable	Factor	Low level (− 1)	High level (+ 1)	Coefficient (coded model)	*t*-value	*P*-value*
X1	ZmPAL (mg/mL)	1.5	2.25	8.35	2.92	0.027
X2	At4CL4 (mg/mL)	1.5	2.25	−2.5	−0.87	0.416
X3	GmCHS (mg/mL)	1.25	1.88	−8.77	−3.06	0.022
X4	MsCHR (mg/mL)	1.25	1.88	7.62	2.66	0.037
X5	ZmCHI (mg/mL)	1	1.50	−4.68	−1.64	0.153
X6	CoA (mM)	1.5	2.25	3.99	1.39	0.213
X7	ATP (mM)	1.5	2.25	0.75	0.26	0.801
X8	NADPH (mM)	1.5	2.25	−5.83	−2.04	0.088
X9	Substrate tyrosine (mM)	2	3	5.73	2	0.092
X10	pH	6	8	7.07	2.47	0.048
X11	T (℃)	30	37	5.7	1.99	0.094
X12	Time (h)	24	36	−1.76	−0.62	0.561
X13	Volume (μL)	50	75	8.66	3.03	0.023

^*^Significant at 95% confidence degree (*P* < 0.05)

### Machine learning experimental design

**Fig. 2:**
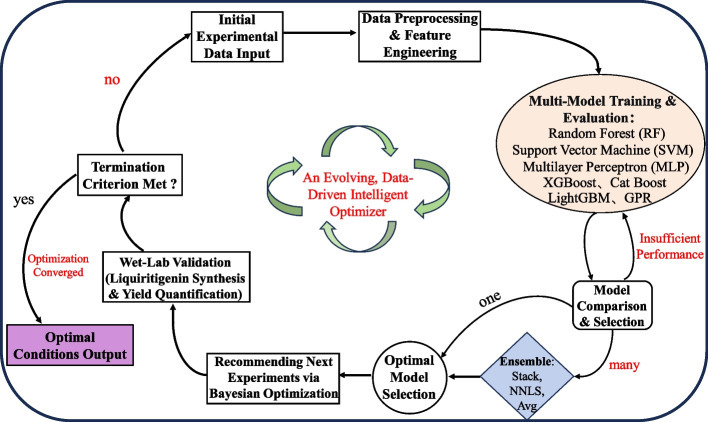
Workflow of the machine learning-driven optimization of cell-free liquiritigenin biosynthesis. The iterative cycle begins with an initial dataset from traditional experimental designs. Data is preprocessed and used to train multiple machine learning models, from which the best performer is selected to guide Bayesian Optimization in proposing the most promising reaction conditions. These conditions are experimentally validated, and the resulting yield data is used to update the dataset and retrain the model, closing the machine learning loop. The process repeats until convergence, outputting the globally optimized biocatalytic process. XGBoost: eXtreme Gradient Boosting; LightGBM: Light Gradient Boosting Machine; CatBoost: Categorical Boosting; GPR: Gaussian Process Regression; NNLS: Non-Negative Least Squares, Avg: Averaging

To overcome the limits of traditional optimization methods, this study developed an active machine learning-driven workflow (Fig. 2) based on prior one-factor-at-a-time, PB, and steepest ascent experiments. The framework uses an iterative cycle of "data input → model training and evaluation → Bayesian optimization → experimental validation" that allows autonomous multi-round optimization until product yield stabilizes. Specifically, based on 13 key parameters identified through preliminary experiments, we first conducted a PB design with 20 experiments to find significant factors, then performed five rounds of steepest ascent experiments to approach the optimal operating region. This process produced 75 experimental points that served as the initial input for machine learning modeling. To handle complex nonlinear relationships among variables and the small sample size of our data, we systematically implemented seven types of machine learning models: classical algorithms (Random Forest, Support Vector Machine, Multilayer Perceptron), advanced ensemble tree methods eXtreme Gradient Boosting (XGBoost), Light Gradient Boosting Machine (LightGBM), Categorical Boosting (CatBoost), and Gaussian Process Regression (GPR), chosen for its suitability for small data. When multiple models demonstrated comparable performance, we integrated all seven using three ensemble methods-Stacking (with RidgeCV as the meta-learner), Simple Averaging (Mean Prediction of the Models) and Non-Negative Least Squares (NNLS) to further enhance predictive accuracy. Model training used a nested cross-validation strategy, with inner three-fold validation for tuning hyperparameters and outer nine-fold validation assessing generalization. The best model based on test-set correlation was selected as the surrogate model for Bayesian optimization. After 2,000 search iterations, the top 12 predicted conditions were experimentally validated. Data from each validation cycle was incorporated into the training set, enabling continuous model improvement and autonomous system optimization.

### Metabolite analysis

Metabolites were analyzed using a Dionex Ultimate 3000 HPLC system following the method of a published protocol (Stahlhut et al. 2015). Separation was performed on a C18 reversed-phase column maintained at 30 °C. The mobile phase consisted of 10 mM ammonium formate (pH 3.0, solvent A) and acetonitrile (solvent B), with the following gradient: 0–0.5 min, 5% B; 0.5–7 min, 5–60% B; 7–9.5 min, 60% B; 9.5–9.6 min, 60–5% B; 9.6–12 min, 5% B. The flow rate was 1.5 mL/min. UV detection was performed at 277, 290, 333, and 370 nm. Retention times under these conditions were: L-tyrosine, 1.8 min (277 nm);* p*-coumaric acid, 6.35 min (333 nm); liquiritigenin, 7.69 min (277 nm); Naringenin Chalcone, 8.17 min (370 nm); naringenin, 8.33 min (290 nm); and isoliquiritigenin, 8.82 min (370 nm) (Figure S1). Standard calibration curves were generated for *p*-coumaric acid, naringenin, liquiritigenin, and isoliquiritigenin, and used for quantitative analysis.

### Statistical analysis

All experiments were performed with three independent biological replicates, and the results were presented as the mean ± standard error (SE). One-way analysis of variance (ANOVA) was employed for statistical analysis of the data, where significance was defined as *P < 0.05, **P < 0.01, ***P < 0.001, and ****P < 0.0001.

## Results and discussion

### Assembly of the in vitro biosynthetic pathway of liquiritigenin

**Fig. 3:**
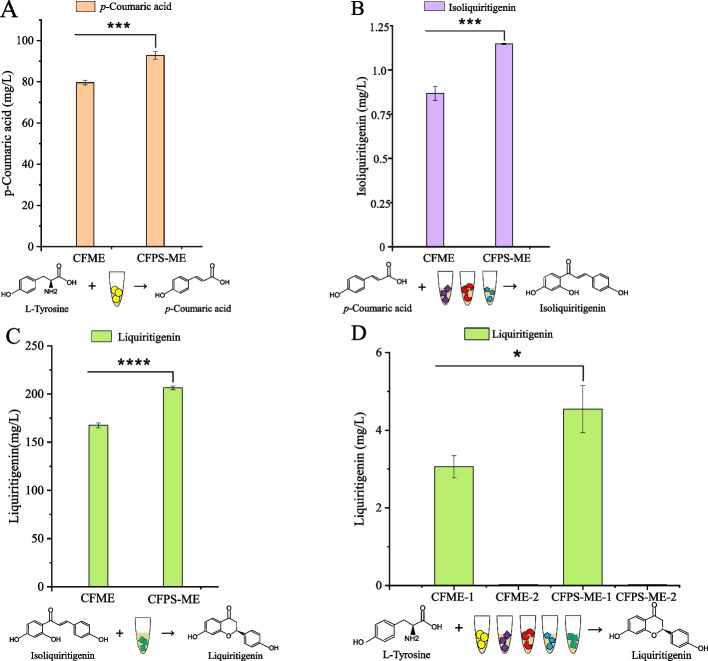
Reconstruction of the liquiritigenin pathway in vitro: comparison of CFME and CFPS-ME methods. **A** Step 1: Conversion of tyrosine to *p*-coumaric acid using ZmPAL. **B** Step 2: Conversion of *p*-coumaric acid to isoliquiritigenin using Pc4CL2, PhCHSA and MsCHR. **C** Step 3: Conversion of isoliquiritigenin to liquiritigenin using MsCHI. **D** Liquiritigenin biosynthesis using ZmPAL, Pc4CL2, PhCHSA, MsCHR and MsCHI from L-tyrosine. Three independent experiments were performed, and the error bars stand for average ± one standard deviation

We firstly compared the effect of CFME and CFPS-ME on the cell-free biosynthesis of liquiritigenin. As shown in Fig. 3, the CFPS-ME system showed higher performance for the biosynthesis of p-coumaric acid, isoliguiritigenin and liquiritigenin than the CFME system. Liquiritigenin was produced 4.55 ± 0.61 mg/L from L-tyrosine using CFPS-ME system.

### Multi-enzyme screening and plasmid dosage optimization in CFPS

**Fig. 4:**
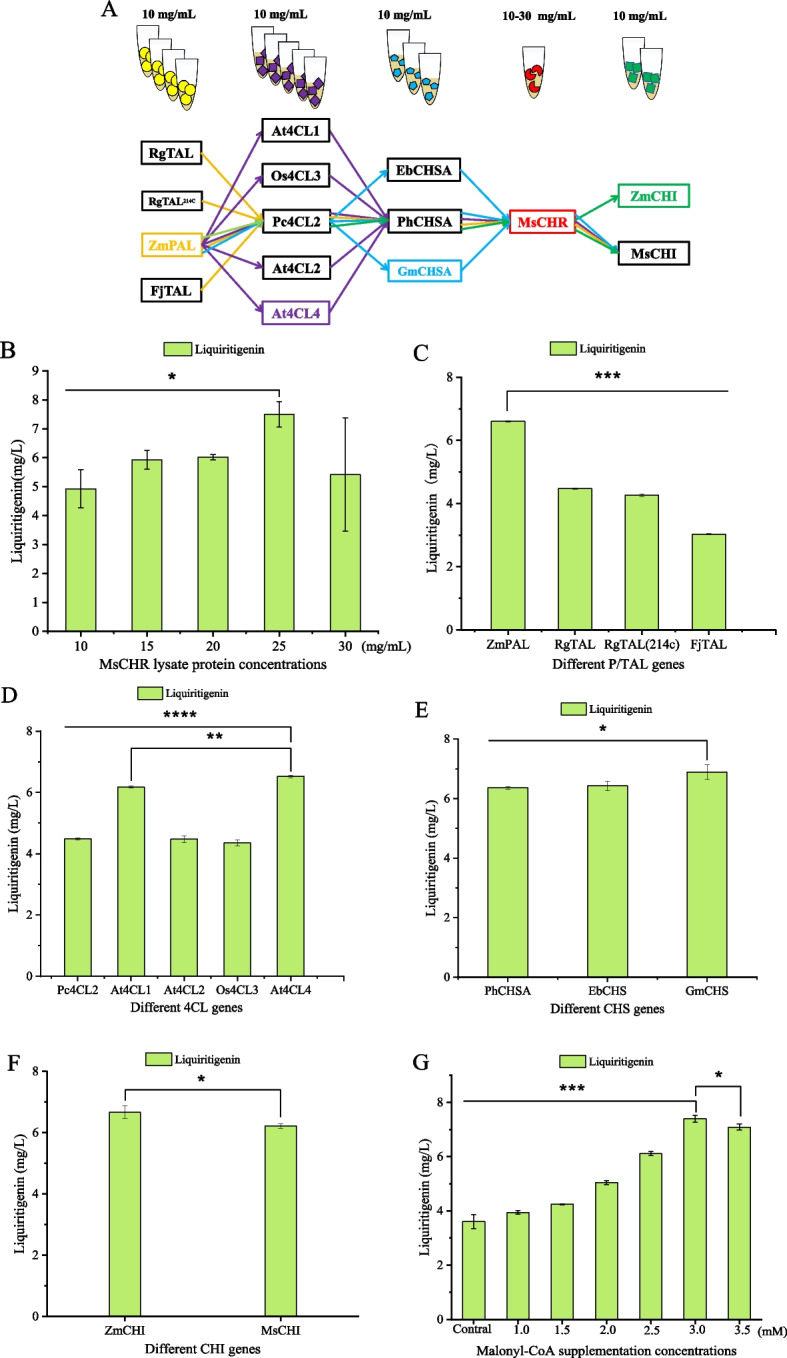
Screening of enzymes for the liquiritigenin biosynthetic pathway using the CFME system with total protein concentrations of 10 mg/mL. **A** Schematic diagram of the multi-enzyme screening approach. **B** Investigation of the effect of MsCHR enzyme concentration. **C**-**F** Screening of individual enzymes: **C** P/TAL, **D** 4CL, **E** CHS, and **F** CHI. **G** Optimization of malonyl-CoA concentration. Three independent experiments were performed, and the error bars stand for average ± one standard deviation

Taking advantage of the rapid and flexible nature of cell-free systems, we systematically screened homologs of the pathway enzymes to enhance liquiritigenin production. Based on the NCBI database and recent literature, candidate isoenzymes and homologs with higher reported catalytic activities were selected (Table 1 and Fig. 4A) and evaluated for their performance in cell-free liquiritigenin synthesis. To establish an efficient biosynthetic pathway, candidate enzymes were first prepared as crude lysates using the CFME system, with total protein concentrations of 10 mg/mL.

Chalcone reductase (CHR) is the key enzyme catalyzing the conversion of p-coumaroyl-CoA to isoliquiritigenin, acting in concert with chalcone synthase (CHS). Adequate malonyl-CoA supply is essential for efficient CHS/CHR activity; insufficient malonyl-CoA decreases CHS efficiency, reduces intermediate accumulation, and indirectly limits CHR activity, thereby favoring naringenin formation. Thus, optimization of CHR protein dosage and malonyl-CoA supplementation was of particular importance. CFME system was used to screen the pathway enzyme and its concentration. And then the plasmid concentrations of the gene of the pathway enzyme were optimized using CFPS-ME system. MsCHR from *M. sativa*, reported as the most efficient CHR in literature (Akram et al. 2021), was selected for detailed evaluation. Overexpression of CHR in the liquiritigenin-producing strain further increased the production of liquiritigenin (Deng et al. 2025). Thus, we investigated the effect of MsCHR concentration on the production of liquiritigenin. The results showed that the yield of liquiritigenin reached a maximum of 7.50 ± 0.44 mg/L when the concentration of MsCHR was 25 mg/mL (Fig. 4B). This suggests that a moderate increase in the amount of MsCHR enzyme is beneficial for product synthesis, whereas excessive MsCHR has an inhibitory effect. Subsequently, the plasmid of MsCHR was optimized using the CFPS system. The results showed that a plasmid concentration of 20 ng/μL yielded the highest production of liquiritigenin (Figure S2). From Fig. 4C-G, we can see that the optimal enzyme combination of ZmPAL, At4CL4, GmCHS, MsCHR, and ZmCHI was obtained. The plasmid concentrations of them in the CFPS system were 35, 15, 20, 20, and 20 ng/μL, respectively (Figure S2).

### Optimization of enzyme ratios and initial substrate concentration

**Fig. 5:**
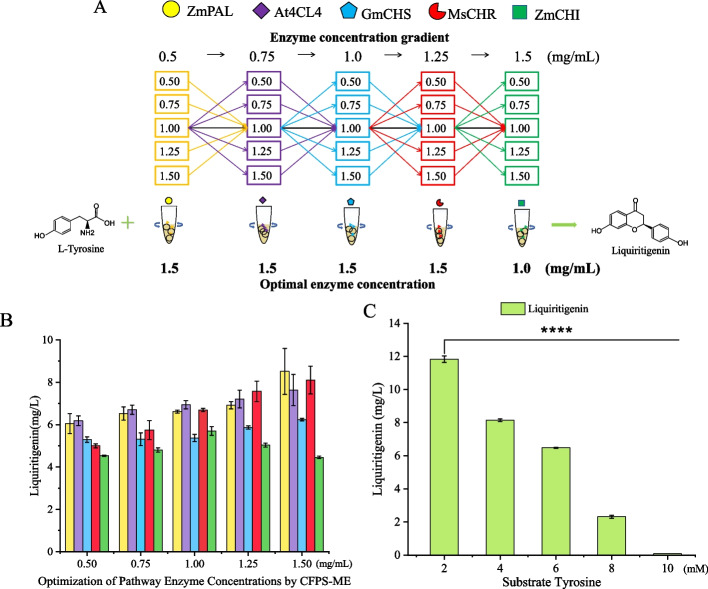
Optimization of enzyme concentration ratios for the optimal enzyme combination in the liquiritigenin biosynthetic pathway using the CFPS-ME system. **A** Liquiritigenin production levels under varying concentrations of the individual enzymes ZmPAL, At4CL4, GmCHS, MsCHR, and ZmCHI. **B** Determination of the optimal protein concentration for each enzyme through univariate screening across a concentration gradient (0.5–1.5 mg/mL). **C** Investigation of the effect of substrate concentration (tyrosine feeding) on production. Three independent experiments were performed, and the error bars stand for average ± one standard deviation

After identifying the optimal enzyme combination for liquiritigenin biosynthesis, we investigated enzyme ratios and initial substrate concentrations using the CFPS-ME system. Cell-free cocktail reactions were used to optimize the enzyme concentration (Fig. 5A). First, ZmPAL protein concentrations were set at 0.5, 0.75, 1.0, 1.25, and 1.5 mg/mL, while the other four enzymes were fixed at 1.0 mg/mL. As shown in Fig. 5B, the highest liquiritigenin yield (8.51 ± 1.09 mg/L) was obtained at ZmPAL of 1.5 mg/mL (*P* < 0.01). Subsequently, the same method was adopted to screen and optimize the concentration ratios of the other four enzymes one after another (Fig. 5A). It can be seen that the highest yield of liquiritigenin was observed at 1.5 mg/mL of ZmPAL (*P* < 0.01), 1.5 mg/mL of At4CL4 (*P* < 0.05), 1.5 mg/mL of GmCHS (*P* < 0.01), 1.5 mg/mL of MsCHR (*P* < 0.01) and 1.0 mg/mL of ZmCHI (*P* < 0.001) (Fig. 5B).

Then the effect of substrate concentration was also investigated. The highest yield was achieved at 2 mM L-tyrosine, whereas concentrations above 6 mM resulted in nearly undetectable product formation (Fig. 5C). This inhibition is likely because the excessive addition of L-tyrosine significantly decreased the pH value.

### Multi-factor optimization of cell-free biosynthesis

**Fig. 6:**
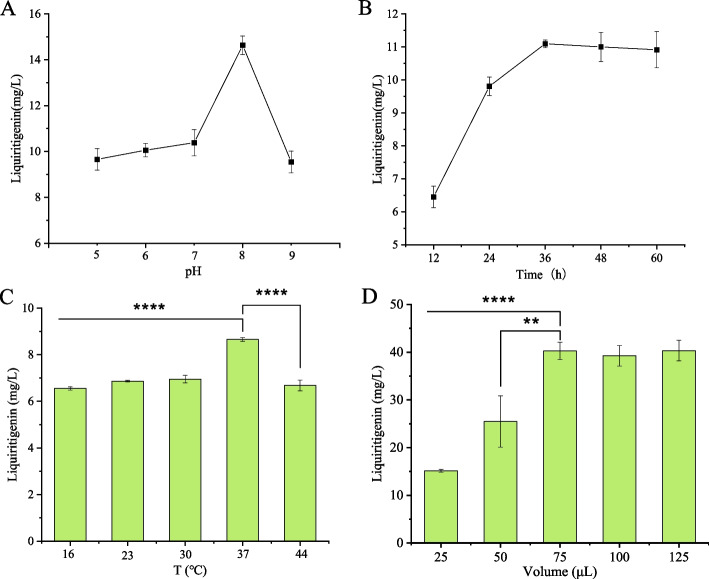
Univariate analysis of reaction conditions for cell-free biosynthesis of liquiritigenin using the CFPS-ME system. **A** Effects of pH on liquiritigenin synthesis. **B** Effects of reaction time on liquiritigenin yield. **C** Effects of reaction temperature on liquiritigenin yield. **D** Effects of reaction volume/system scale on liquiritigenin yield. Three independent experiments were performed, and the error bars stand for average ± one standard deviation

We then investigated the effects of several key parameters on the production of liquiritigenin. We first examined the effect of reaction temperature by setting gradients of 16, 23, 30, 37, and 44 °C. The results showed that the biosynthesis efficiency of liquiritigenin was highest at 37 °C, reaching a yield of 8.65 ± 0.08 mg/L (Fig. 6C). Subsequently, pH values (5, 6, 7, 8, 9), reaction times (12, 24, 36, 48, 60 h), and reaction volumes (25, 50, 75, 100, 125 μL) were optimized. The results showed that the highest production of liquiritigenin was obtained at a reaction time of 36 h, a temperature of 37 °C, pH 8.0 and the reaction volume of 75 μL (Fig. 6A, B, D).

A Plackett–Burman experiment was then employed to further optimize the CFBS of liquiritigenin. The experimental design and corresponding results are presented in Tables 2 and 3. Experiment No. 18 achieved the highest yield of 79.64 ± 1.95 mg/L.

**Table 3:** Level code for each variable in the Plackett–Burman design and the response of liquiritigenin

Run	Variable levels													Liquiritigenin (mg/L)
	X1	X2	X3	X4	X5	X6	X7	X8	X9	X10	X11	X12	X13	
1	−1	1	−1	1	−1	1	1	1	1	−1	−1	1	1	50.43 ± 0.05
2	1	1	−1	1	1	−1	−1	−1	−1	1	−1	1	−1	52.41 ± 1.96
3	1	1	−1	−1	1	1	−1	1	1	−1	−1	−1	−1	14.24 ± 1.81
4	−1	−1	−1	1	−1	1	−1	1	1	1	1	−1	−1	76.42 ± 2.82
5	−1	−1	−1	−1	1	−1	1	−1	1	1	1	1	−1	37.36 ± 5.58
6	1	1	1	−1	−1	1	1	−1	1	1	−1	−1	−1	65.37 ± 3.20
7	−1	1	1	−1	1	1	−1	−1	−1	−1	1	−1	1	17.45 ± 2.40
8	−1	−1	1	1	−1	1	1	−1	−1	−1	−1	1	−1	13.96 ± 0.71
9	−1	−1	1	−1	1	−1	1	1	1	1	−1	−1	1	20.86 ± 4.09
10	−1	1	1	−1	−1	−1	−1	1	−1	1	−1	1	1	11.41 ± 0.92
11	1	1	1	1	−1	−1	1	1	−1	1	1	−1	−1	26.45 ± 1.04
12	−1	−1	−1	−1	−1	−1	−1	−1	−1	−1	−1	−1	−1	15.60 ± 2.24
13	1	−1	1	1	−1	−1	−1	−1	1	−1	1	−1	1	78.59 ± 8.76
14	1	−1	1	1	1	1	−1	−1	1	1	−1	1	1	66.55 ± 2.24
15	1	1	−1	−1	−1	−1	1	−1	1	−1	1	1	1	71.43 ± 2.81
16	−1	1	1	1	1	−1	−1	1	1	−1	1	1	−1	15.94 ± 2.37
17	1	−1	1	−1	1	1	1	1	−1	−1	1	1	−1	17.81 ± 0.46
18	−1	1	−1	1	1	1	1	−1	−1	1	1	−1	1	79.64 ± 1.95
19	1	−1	−1	1	1	−1	1	1	−1	−1	−1	−1	1	58.01 ± 4.77
20	1	−1	−1	−1	−1	1	−1	1	−1	1	1	1	1	75.77 ± 5.78

The data represent the means of three replicates, and error bars represent standard deviations

The following first-order equation was found to explain the liquiritigenin production (Y) after regression analysis:

$$\mathrm Y\;=\;38.54\;+\;8.35{\mathrm X}_1\;-\;2.50{\mathrm X}_2\;-\;8.77{\mathrm X}_3\;+\;7.62{\mathrm X}_4\;-\;4.68{\mathrm X}_5\;+\;3.99{\mathrm X}_6\;+\;0.75{\mathrm X}_7\;-\;5.83{\mathrm X}_8\;+\;5.73{\mathrm X}_9\;+\;7.07{\mathrm X}_{10}\;+\;5.70{\mathrm X}_{11}\;-\;1.76{\mathrm X}_{12}\;+\;8.66{\mathrm X}_{13}$$

The coefficients in this equation reflect the influence of each variable on the liquiritigenin production. Table 1 shows the statistical analysis of each factor. Results demonstrated that ZmPAL (X₁), GmCHS (X₃), MsCHR (X₄), pH (X₁₀), and reaction volume (X₁₃) had a significant effect on liquiritigenin production. ZmPAL (X₁), GmCHS (X₃), and MsCHR (X₄) were identified as the key enzymes in the biosynthesis pathway, while pH (X₁₀) and reaction volume (X₁₃) were the major environmental factors.

Moreover, the equation revealed that coefficients of X₁ (ZmPAL), X₄ (MsCHR), X₁₀ (pH), and X₁₃ (volume) were positive, while X₃ (GmCHS) was negative. It indicates that increasing concentrations of ZmPAL and MsCHR, enlarging the reaction volume, and slightly elevating pH were favorable for liquiritigenin production, whereas decreasing GmCHS concentration had a positive effect. Therefore, the steepest ascent method was employed to optimize these five variables (pH, volume, ZmPAL, GmCHS, MsCHR). Table 4 summarizes the design and corresponding results. After the steepest ascent experiments, the final liquiritigenin yield reached 104.42 ± 1.51 mg/L.

**Table 4:** Experimental design of the steepest ascent and the corresponding liquiritigenin yield

Trial	MsCHR (mg/mL)	ZmPAL (mg/mL)	GmCHS (mg/mL)	volume (μL)	pH	Liquiritigenin (mg/L)
1	1.88	2.25	1.25	50	7.0	76.20 ± 4.35
2	2.26	2.70	1.0	75	7.5	93.63 ± 8.47
3	2.63	3.15	0.75	100	8.0	104.42 ± 1.51
4	3.0	3.60	0.5	125	8.5	97.64 ± 3.42
5	3.38	4.05	0.25	150	9.0	76.46 ± 4.31

The data represent the means ± standard deviations (s.d.) of three replicates

### Machine learning for reaction system optimization

**Fig. 7:**
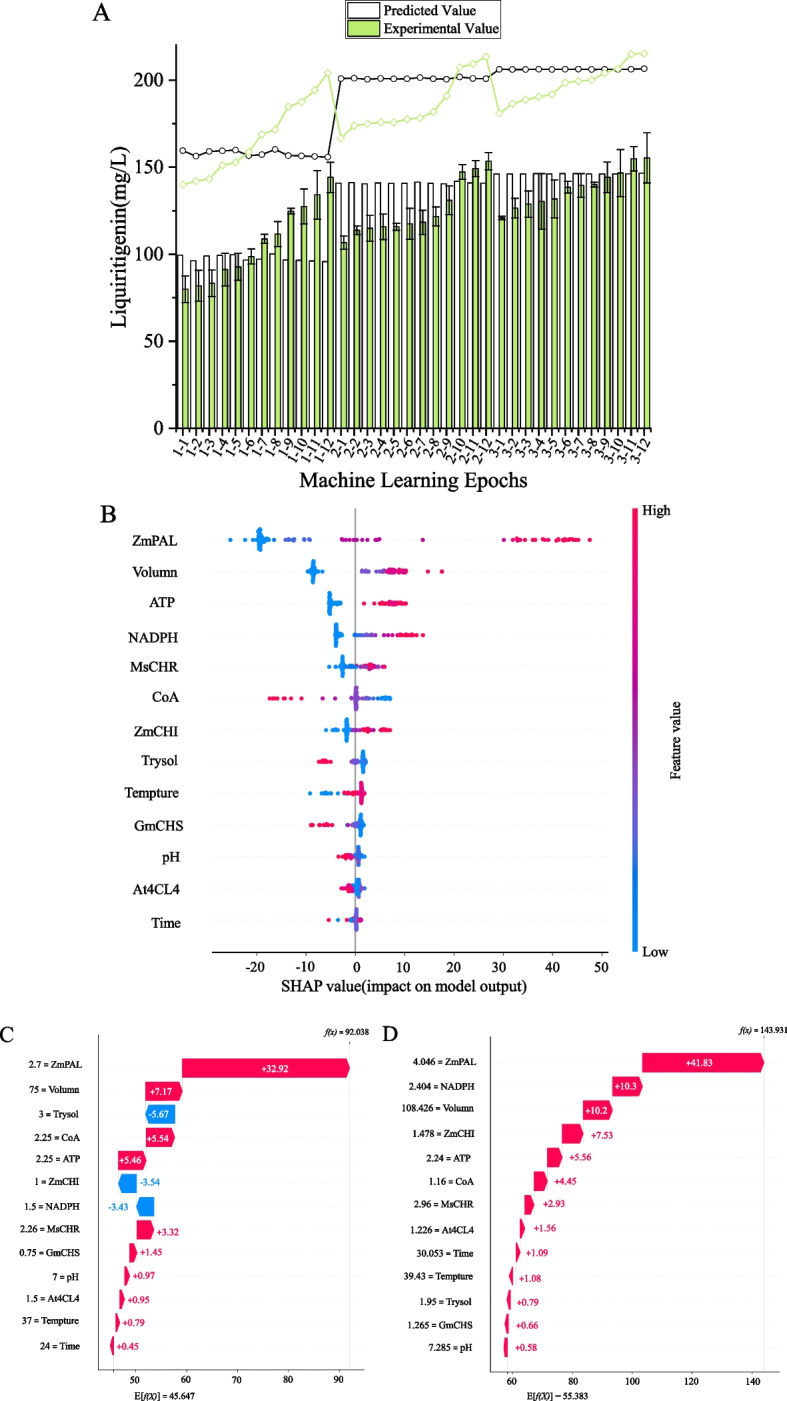
Evaluation of the machine learning model predictions through iterative experimental validation. **A** Comparison of the model-predicted yields against the actual experimental measurements across all three iterative rounds, showing the overall convergence trend. Black lines and white bars represent the predicted trends and yields, respectively; green lines and bars correspond to the experimentally measured values. The mean value of the third round approaches the predicted level. The sequential increase in the mean and reduction in variance across rounds indicate a converging optimization process. Three independent experiments were performed, and the error bars stand for average ± one standard deviation. **B** SHAP summary plot illustrating feature impacts on the target output. Red hues indicate features with a positive correlation to liquiritigenin yield, whereas blue hues represent a negative correlation. The horizontal dispersion of points for a feature reflects the magnitude of its impact, with values further from zero indicating stronger effects. **C** Waterfall plot from the first prediction round, depicting the cumulative contribution of each input feature (from the base value) to the predicted output for a specific instance. Features increasing the prediction are shown in red, and those decreasing it are in blue. **D** Waterfall plot corresponding to the best-performing prediction round, demonstrating the refined feature contributions in the final optimization cycle

Following the Plackett–Burman (PB) and steepest ascent experiments, we integrated the resulting data with prior single-factor and related experimental datasets to further optimize the reaction system using a machine learning workflow incorporating multiple machine learning models. Through three iterative rounds of machine learning, the production of liquiritigenin was significantly enhanced. As shown in Fig. 7A, among the top 12 performing combinations selected from each round of wet-lab experimentation, the average yield increased progressively from 106.55 ± 21.05 mg/L in the first round to 125.46 ± 15.20 mg/L in the second, and further to 138.11 ± 10.45 mg/L in the third. This trend not only demonstrates a consistent upward trajectory in the mean yield but also reveals a continual reduction in both inter-group variability and variance. Correspondingly, the average error between the model-predicted values and the actual experimental measurements decreased significantly. Furthermore, the test *R*^2^ values obtained during model training showed clear improvement (Table S3). In the final modeling round, three ensemble strategies (Stacking, Simple Averaging, and NNLS-weighted fusion) were evaluated. The Stacking approach, which utilized RidgeCV as the meta-learner in the second layer, delivered the best performance, achieving a test *R*^2^ of 0.903. This result indicates that the final model exhibits excellent overall fit to the dataset and is capable of making accurate predictions of experimental outcomes. SHapley Additive exPlanations (SHAP) analysis confirmed that fine-tuning the enzyme ratios and cofactor concentrations within the reaction system substantially enhanced liquiritigenin production in the cell-free system. SHAP summary plot analysis (Fig. 7B) identified the enzyme ZmPAL, reaction volume, ATP, and NADPH as the most influential features on the model output. Among them, ZmPAL exhibited a strong positive correlation, indicating that higher enzyme concentrations led to increased predicted yields. Similarly, reaction volume, ATP, and NADPH demonstrated positive correlations. In contrast, CoA, the substrate tyrosine, and GmCHS displayed negative correlations, suggesting that higher values for these factors inversely affected the predicted yield.

The SHAP waterfall plot (Fig. 7C) illustrates the results of the first prediction round, based on conditions from the PB and steepest ascent experiments. Features colored red acted as the primary drivers, increasing the predicted output for this sample. Specifically, ZmPAL contributed the most substantial positive influence (+ 32.92), elevating the predicted value by approximately 35.8%. Reaction volume, CoA and ATP ranked as the second and third most significant positive factors, increasing the prediction score by 7.17, 5.54 and 5.46, respectively. While features colored blue (substrate tyrosine, ZmCHI, and NADPH) exerted inhibitory effects, their overall impact was comparatively minor against the combined positive drive of the red features. The SHAP waterfall plot in Fig. 7D depicts the optimal reaction conditions for cell-free liquiritigenin biosynthesis, as predicted by the model in the third optimization round. Under these conditions, the system achieved a yield of 155.32 ± 14.39 mg/L, representing a 48.7% increase over the results from the initial Plackett–Burman and steepest ascent experiments and a more than twofold improvement in conversion rate. Consequently, the optimized reaction conditions adopted for subsequent studies were as follows: ZmPAL 4 mg/mL; At4CL4 1.2 mg/mL; GmCHS 1.3 mg/mL; MsCHR 3 mg/mL; ZmCHI 1.5 mg/mL; 2.2 mM ATP; 1.2 mM CoA; 2.4 mM NADPH; 2 mM tyrosine; 8 mM Mg(CH₃COO)₂·4H₂O; 4 mM CH₃COONH₄; 134 mM CH₃COOK; 10 mM K₂HPO₄; 3.0 mM malonyl-CoA; 108 μL reaction volume; pH 7.3; 39 °C; 30 h reaction time.

### Multi-enzyme assembly and application of protein scaffolds

**Fig. 8:**
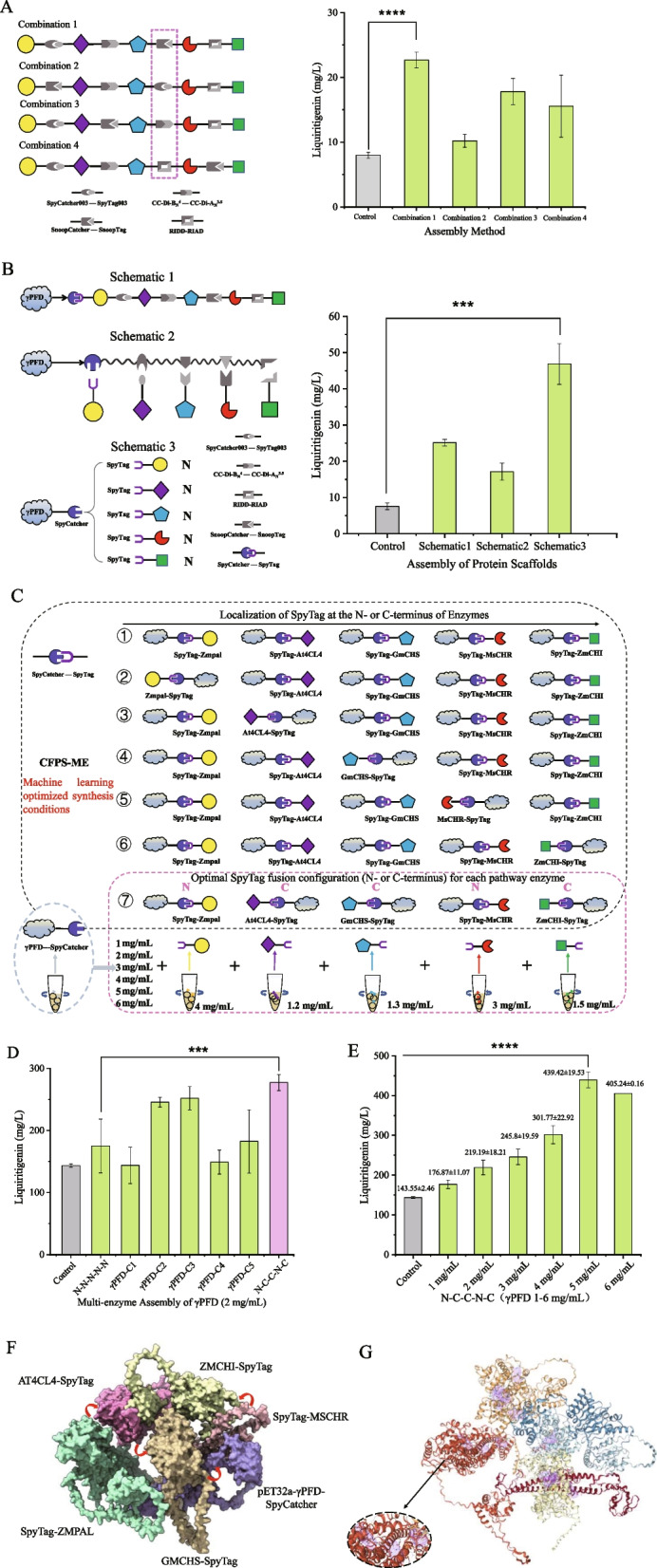
Biosynthesis of liquiritigenin using a protein scaffold-based self-assembly system combined with the cell-free platform. **A** Liquiritigenin production yields achieved by four distinct covalent peptide-mediated multi-enzyme assembly strategies using the CFME system. **B** The effect of the γPFD-SpyCatcher protein scaffold on liquiritigenin biosynthesis yield when combined with three multi-enzyme assembly strategies, identifying γPFD-SpyCatcher-pathway enzyme-SpyTag as the optimal combination using the CFME system. **C** Schematic of the experimental scheme to investigate the interaction of γPFD-SpyCatcher with pathway enzymes carrying N- or C-terminal SpyTag. **D** Comparison of liquiritigenin production upon binding of γPFD-SpyCatcher to pathway enzymes fused with SpyTag at the N- or C-terminus using the CFPS-ME system under machine learning-optimized synthesis conditions. **E** Liquiritigenin production at varying γPFD-SpyCatcher concentrations (1–6 mg/mL) under the optimal (N–C-C-N–C) binding configuration using the CFPS-ME system under machine learning-optimized synthesis conditions. **F** Model of the γPFD–enzyme complex assembled by SpyTag/SpyCatcher. Red arrows trace the reaction sequence. The raw predicted models were further subjected to structural visualization, refinement, and figure preparation using UCSF ChimeraX. **G** Architecture of the catalytic sites in the complex. Active pockets are shown in purple (https://proteins.plus). Three independent experiments were performed, and the error bars stand for average ± one standard deviation

Five pathway enzymes were first assembled within the CFME system using four pairs of self-assembling covalent peptides: SnoopTag/SnoopCatcher, SpyTag003/SpyCatcher003, CC-Di-AN35/CC-Di-BN40, and RIAD/RIDD. The effects of these four covalent peptide pairs were particularly examined between GmCHS and MsCHR, designing four different combinations (Fig. 8A) to evaluate multi-enzyme assembly and investigate how specific peptide pairs could enhance catalytic synergy between the two enzymes. SDS-PAGE analysis (Figure S3) confirmed the expression of the target protein in each crude enzyme lysate. In the CFME system with each enzyme at 10 mg/mL, assembly was observed to enhance the production of liquiritigenin (Fig. 8A). Combination 1 exhibited the best performance with producing 22.69 ± 1.23 mg/L liquiritigenin.

Subsequently, a protein scaffold system was introduced to enhance enzymatic interactions and thereby improve catalytic efficiency. To this end, this study aimed to develop a simple and efficient self-assembling protein scaffold for the immobilization of multiple enzymes in vitro. We selected eight scaffold proteins: ethanolamine utilization microcompartment shell protein (EutM) from *Salmonella enterica*, Filamentous chaperone protein (γ-prefoldin, γ-PFD) from hyperthermophilic archaea, Amyloid fibril protein (Sup35^1−61^) from *Saccharomyces cerevisiae*, capsid protein (P22CP) from bacteriophage P22, capsid protein (MS2) from bacteriophage MS2, major envelope protein (P9) from bacteriophage ϕ6, SARS coronavirus envelope protein (SCVE), hepatitis B virus core antigen (HBcAg). These were assembled with ZmPAL using the SpyTag/Catcher system, followed by the addition of substrate to initiate the synthesis of *p*-coumaric acid. It can be seen that assembling γPFD-SpyCatcher with SpyTag-ZmPAL exhibited the highest conversion efficiency of *p*-coumaric acid (Figure S4). Then, three strategies were employed to assemble these five pathway enzymes with the optimal scaffold γPFD-SpyCatcher (Fig. 8B). Strategy 1: The CFME system contained γPFD-SpyCatcher, SpyTag-ZmPAL-SpyCatcher003, SpyTag003-At4CL4-CCDIA, CCDIB-GmCHS-SnoopTag, SnoopCatcger-MsCHR-RIAD, RIDD-ZmCHI. Strategy 2: The system contained γPFD-SpyCatcher-SpyCatcher003-CCDiA-SnoopCatcher-RIAD, SpyTag-ZmPAL, SpyTag003-At4CL4, CCDIB-GmCHS, SnoopTag-MsCHR, RIDD-ZmCHI. Strategy 3: The system contained γPFD-SpyCatcher, SpyTag-ZmPAL, SpyTag-At4CL4, SpyTag-GmCHS, SpyTag-MsCHR, SpyTag-ZmCHI. It can be seen that all of three strategies enhanced the production of liquiritigenin using the CFME system (Fig. 8B). Strategy 3 showed the highest level of liquiritigenin production with 46.89 ± 5.66 mg/L, which is about twice that of no protein scaffold. To further enhance the production of liquiritigenin, we utilized different interactions to assemble the five pathway enzymes to the γPFD scaffold (Fig. 8C). As shown in Fig. 8D, the N–C-C-N–C assembling exhibited the highest level of liquiritigenin production using the CFPS-ME system under the above optimal conditions. We also investigated the effect of the concentration of γPFD scaffold on the production of liquiritigenin. Increasing the concentration of γPFD scaffold enhanced the production of liquiritigenin (Fig. 8E). The highest level of liquiritigenin production (439.42 ± 19.53 mg/L) was obtained using 5 mg/mL γPFD scaffold protein.

Concurrently, structural prediction of the multi-enzyme complex via AlphaFold 3 (Abramson et al. 2024) revealed a compact, prism-like assembly (Fig. 8F), whose catalytic efficiency was enhanced by the close spatial proximity mediated by γPFD and SpyTag/SpyCatcher interactions. Active site prediction via ProteinsPlus (Ehrt et al. 2025) confirmed that strategic SpyTag placement exposed all catalytic pockets (Fig. 8G). This setup facilitates substrate binding and, combined with γPFD, promotes an efficient substrate channel. This channel directs intermediates between active sites, minimizing diffusion and significantly boosting overall efficiency.

**Fig. 9:**
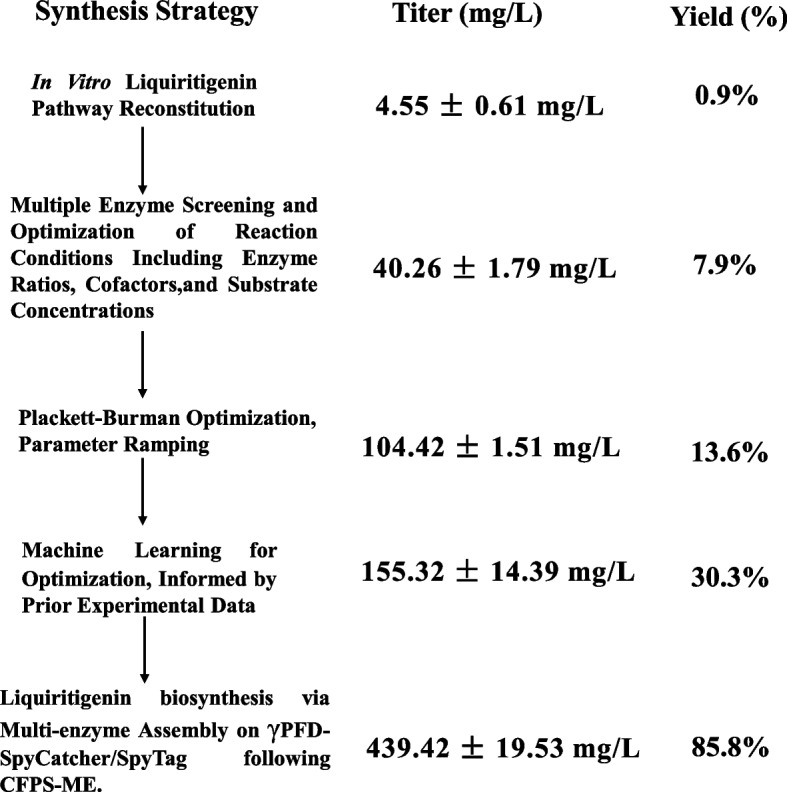
Diagram summarizing steps in this study for liquiritigenin production. γPFD: γ-prefoldin from hyperthermophilic archaea; CFPS-ME: cell-free biosynthesis-metabolic engineering

In this study, a cell-free system for the production of liquiritigenin was developed by combining machine learning-driven optimization with spatial assembly. The liquiritigenin yield was gradually improved through multiple synthetic strategies (Fig. 9). As a result, the yield of liquiritigenin in the CFPS-ME system significantly increased from an initial 4.55 ± 0.61 mg/L to 155.32 ± 14.39 mg/L after the machine learning-driven optimization. Subsequently, the use of self-assembling peptide pairs and protein scaffolds enabled spatially organized enzyme assembly. Four combinations of five covalent peptide pairs were compared under the CFME strategy, and eight protein scaffolds were evaluated for their ability to enhance the enzymatic activity of pathway enzymes. Assembling the five pathway enzymes to the γPFD scaffold protein further enhanced the production of liquiritigenin to 439.42 ± 19.53 mg/L. A conversion rate of 85.8% was achieved at a substrate concentration of 2 mM. The titer and yield of liquiritigenin were increased by 2.83-fold after the spatial assembly.

## Conclusion

A cell-free multienzyme system for efficient production of liquiritigenin was developed. Using CFME and CFPS-ME approach, the optimal enzyme combination (ZmPAL, At4CL4, GmCHS, MsCHR, ZmCHI) was identified through systematic screening and ratio optimization. Three rounds of iterative machine learning, informed by Plackett–Burman and steepest ascent experiments, yielded 155.32 ± 14.39 mg/L of liquiritigenin under optimized conditions. Spatial enzyme assembly via covalent peptide tags and scaffold proteins (γPFD-SpyCatcher) further enhanced the production of liquiritigenin. Spatial assembly enhanced the production of liquiritigenin by 2.83-fold. This study demonstrates that machine learning-driven optimization and spatial assembly of multienzyme is a powerful approach for cell-free biosynthesis.

## Supplementary Information

Supplementary Material 1.

## Data Availability

Data sharing not applicable to this article as no datasets were generated or analyzed during the current study.
