# Supplementary material for: Machine learning-driving optimization and spatial assembly of a cell-free system for high-yield liquiritigenin production
Source: Adv Biotechnol (Singap). 2026 Mar 27;4(2):12. doi: 10.1007/s44307-026-00103-0 (PMC13031608; doi:10.1007/s44307-026-00103-0)
Supplement: Supplementary file 1 — Supplementary Material 1. [file 44307_2026_103_MOESM1_ESM.pdf]

Advanced Biotechnology

Supplemental information

Machine learning-driving optimization and spatial assembly of a cell-free system for high-yield liquiritigenin production

Fei Liu[1], Si-Bo Zhao[1], Yan-Hua Liu[1], Jun-Feng Li[1], Nuo-Qiao Lin[1], Meihereayi Mutailifu[1], Pei Xu[1], and Jian-Zhong Liu[1]*

*Correspondence:

Jian-Zhong Liu

lssljz@mail.sysu.edu.cn

[1] State Key Laboratory of Biocontrol, School of Life Sciences, Sun Yat-Sen University, Guangzhou 510275, People's Republic of China

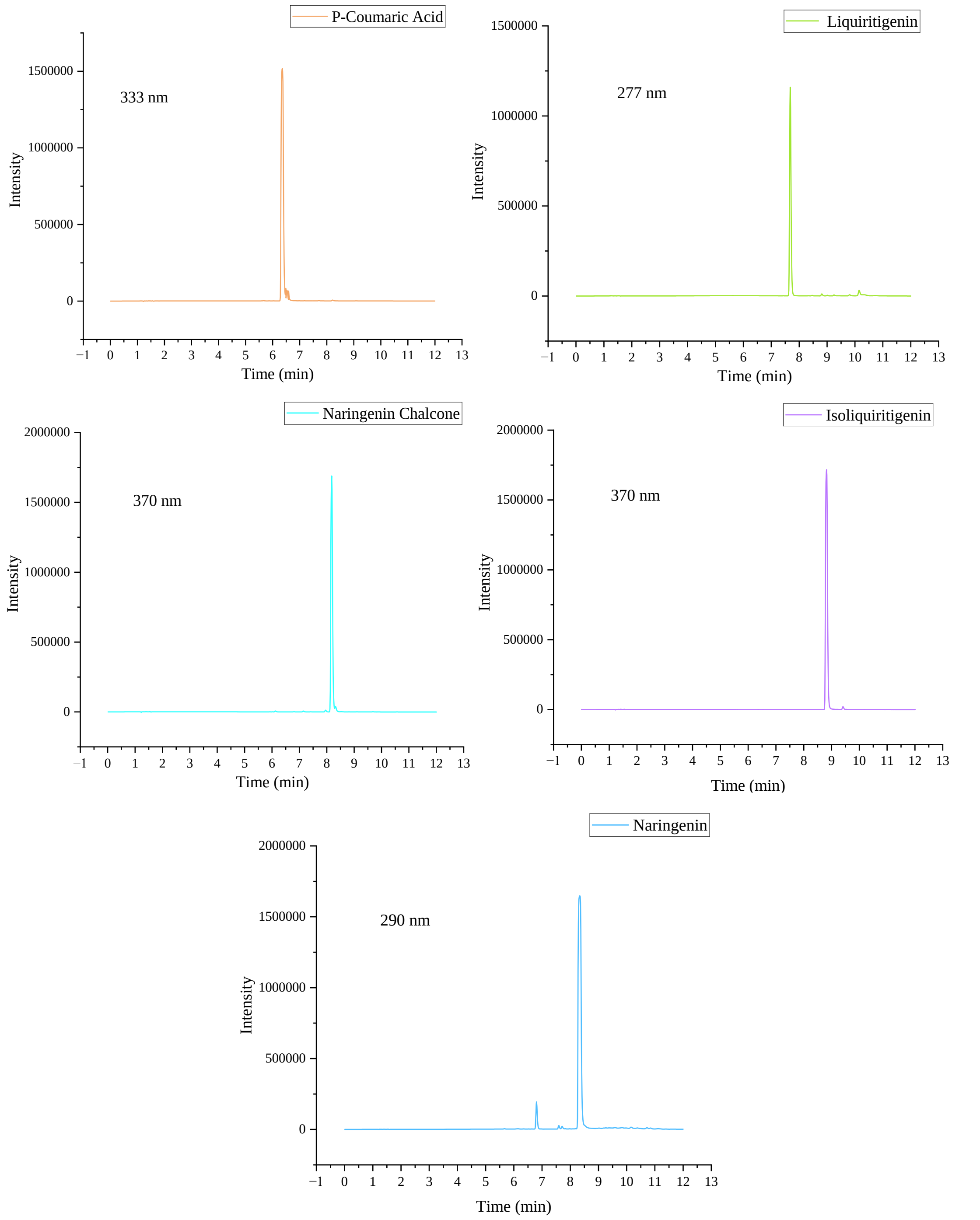

Figure S1. High-performance liquid chromatography (HPLC). *p*-Coumaric acid, 6.35 min (333 nm); Liquiritigenin, 7.69 min (277 nm); Naringenin chalcone, 8.17 min (370 nm); Naringenin, 8.33 min (290 nm); and Isoliquiritigenin, 8.82 min (370 nm). The column temperature was set at 37°C.

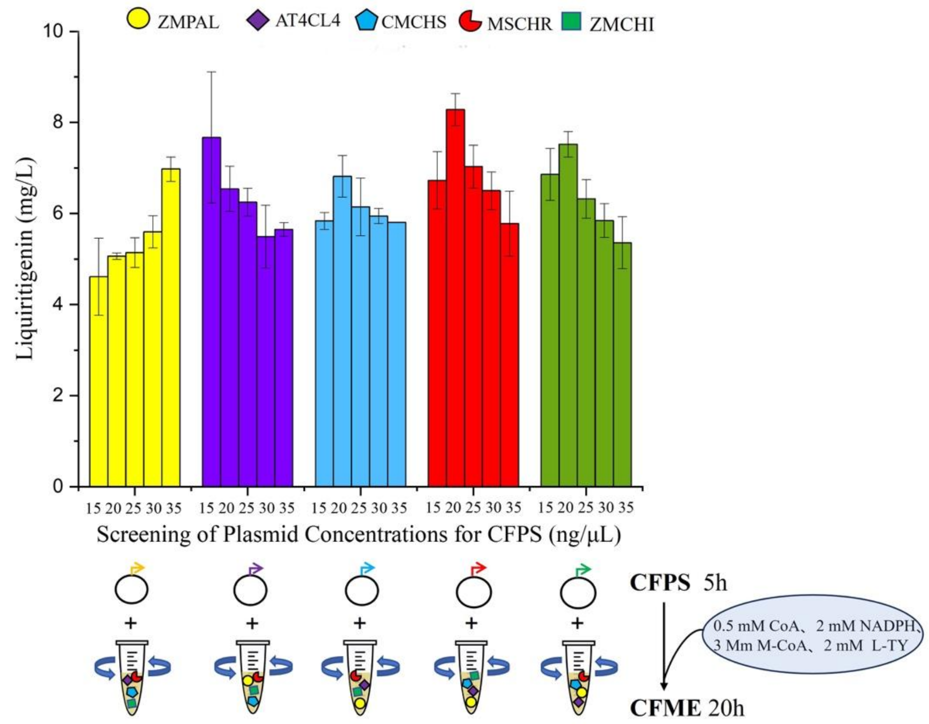

Figure S2. To investigate the effect of varying plasmid concentrations (15-35 ng/μL) for the CFPS of MsCHR, ZmPAL, At4CL4, GmCHS, and ZmCHI on the subsequent CFME production of liquiritigenin, the template plasmid for each enzyme was added to the CFPS reaction mixture and combined with the crude lysates of the other four enzymes (10 mg/mL). Following a 5-hour CFPS reaction at 30°C, the reaction was supplemented with 0.5 mM CoA, 2 mM $NADP^+$, 3 mM malonyl-CoA, and 2 mM L-tyrosine to initiate the synthesis of liquiritigenin at 37°C.

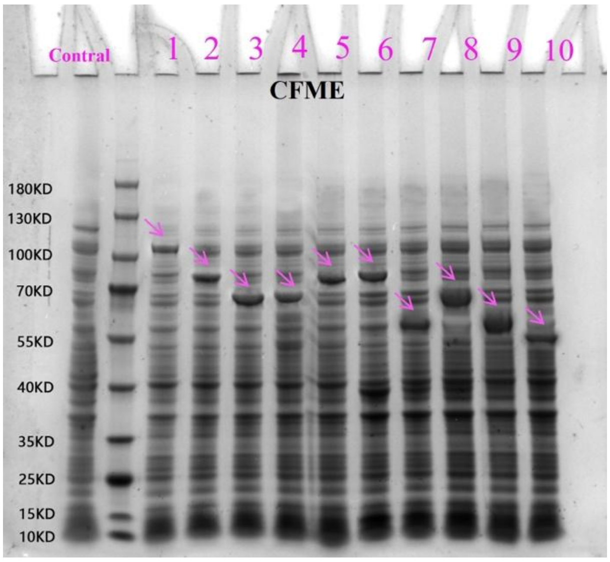

Gene	kDa	Arrow indicates	Gene	kDa	Arrow indicates	Gene	kDa	Arrow indicates
ZmPAL-SnoopCatcher	105	1	SpyTag003--At4CL4--SnoopTag	75.3	5	CCDIB--GmCHS-RIAD	67	8
SnoopTag-At4CL4-CCDIA	77.8	2	SnoopCatcher--GmCHS-CCDIA	77.1	6	RIDD-MsCHR-SnoopTag	61.5	9
CCDIB-GmCHS-SpyTag003	66.6	3	CCDIB-MsCHR-RIAD	59.6	7	SnoopCatcher-ZmCHI	55.6	10
SpyCatcher003-MsCHR-RIAD	69	4	/	/	/	/	/	/

Figure S3. The expression of the five pathway enzymes, fused to four self-assembling covalent peptide pairs (SnoopTag/SnoopCatcher, SpyTag003/SpyCatcher003, CC-Di-AN35/CC-Di-BN40, and RIAD/RIDD) in four distinct assembly modes, was verified by SDS-PAGE analysis of the crude enzyme lysates.

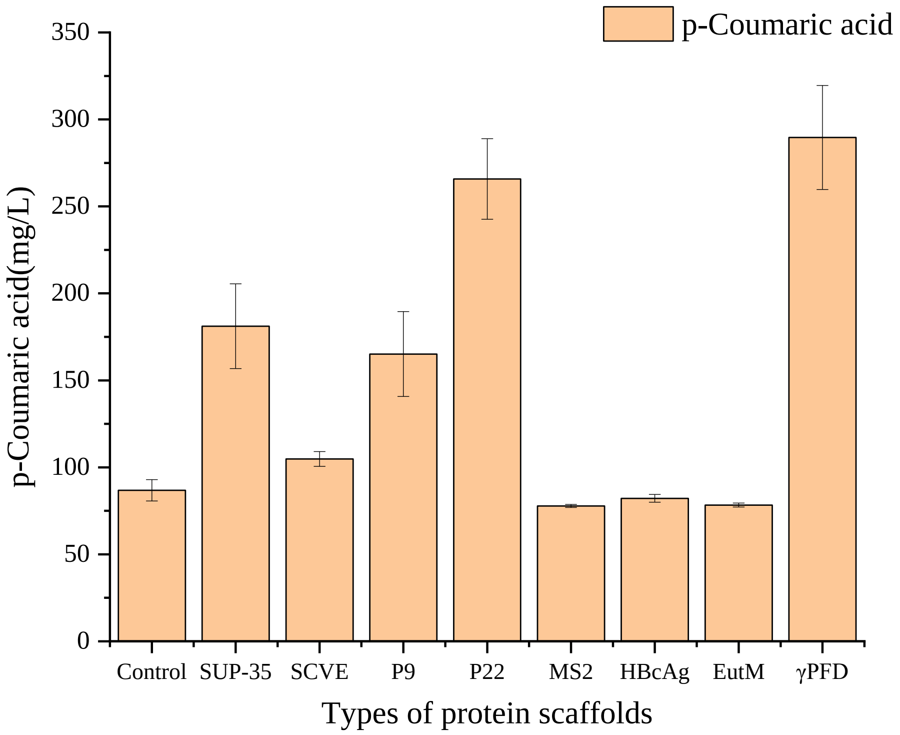

Figure S4. A screening of eight known protein scaffolds (Sup35-SpyCatcher, SCVE-SpyCatcher, P9-SpyCatcher, P22-SpyCatcher, MS2-SpyCatcher, HBcAg-SpyCatcher, EutM-SpyCatcher, and γPFD-SpyCatcher) was conducted by assessing the production of *p*-coumaric acid following their assembly with SpyTag-ZmPAL.

Table S1. Strains and plasmids used in this study

Strains & Plasmids	Relevant characteristics	Source or reference
pET32a-ZmPAL	Plasmid used for CFPS containing ZmPAL	this study
pET32a-Pc4CL2	Plasmid used for CFPS containing Pc4CL2	this study
pET32a-PhCHSA	Plasmid used for CFPS containing PhCHSA	this study
pET32a-MsCHI	Plasmid used for CFPS containing MsCHI	this study
pET32a-MsCHR	Plasmid used for CFPS containing MsCHR	this study
pET32a-At4CL4	Plasmid used for CFPS containing At4CL4	this study
pET32a-GmCHS	Plasmid used for CFPS containing GmCHS	this study
pET32a-ZmCHI	Plasmid used for CFPS containing ZmCHI	this study
pET32a-ZmPAL-Pc4CL2-Phchsa-MsCHR-MsCHI	Plasmid used for CFPS containing ZmPAL-Pc4CL2- Pc4CL2-MsCHR-MsCHI（Restriction-ligation cloning）	this study
pET32a-SpyTag-ZmPAL	Based on the pET32a vector, encoding a ZmPAL with an N-terminal SpyTag fusion.	this study
pET32a-SpyTag-At4CL4	Based on the pET32a vector, encoding a At4CL4 with an N-terminal SpyTag fusion.	this study
pET32a-SpyTag-GmCHS	Based on the pET32a vector, encoding a GmCHS with an N-terminal SpyTag fusion.	this study
pET32a-SpyTag-MsCHR	Based on the pET32a vector, encoding a MsCHR with an N-terminal SpyTag fusion.	this study
pET32a-SpyTag-ZmCHI	Based on the pET32a vector, encoding a ZmCHI with an N-terminal SpyTag fusion.	this study
pET32a-ZmPAL-SpyTag	Based on the pET32a vector, encoding a ZmPAL with an N-terminal SpyTag fusion.	this study

pET32a-At4CL4-SpyTag	Based on the pET32a vector, encoding a At4CL4 with an C-terminal SpyTag fusion.	this study
pET32a-GmCHS-SpyTag	Based on the pET32a vector, encoding a GmCHS with an C-terminal SpyTag fusion.	this study
pET32a-MsCHR-SpyTag	Based on the pET32a vector, encoding a MsCHR with an C-terminal SpyTag fusion.	this study
pET32a-ZmCHI-SpyTag	Based on the pET32a vector, encoding a ZmCHI with an C-terminal SpyTag fusion.	this study
pET32a-Sup35-SpyCatcher	pET32a containing Sup35-SpyCatcher	this study
pET32a-SCVE-SpyCatcher	pET32a containing SCVE-SpyCatcher	this study
pET32a-P9-SpyCatcher	pET32a containing P9-SpyCatcher	this study
pET32a-P22-SpyCatcher	pET32a containing P22-SpyCatcher	this study
pET32a-MS2-SpyCatcher	pET32a containing MS2-SpyCatcher	this study
pET32a- HBcAg-SpyCatcher	pET32a containing HBcAg-SpyCatcher	this study
pET32a-EutM-SpyCatcher	pET32a containing EutM-SpyCatcher	this study
pET32a-γPFD-SpyCatcher	pET32a containing γPFD-SpyCatcher	this study
pET32a-ZmPAL-SpyCatcher003	used for protein production and extract preparation of ZmPAL-SpyCatcher003	this study
pET32a-SPyTag003-At4CL4-CCDIA	used for protein production and extract preparation of SPyTag003-At4CL4-CCDIA	this study
pET32a-CCDIB-GmCHS-SnoopTag	used for protein production and extract preparation of CCDIB-GmCHS-SnoopTag	this study
pET32a-SnoopCatcher-MsCHR-RIAD	used for protein production and extract preparation of SnoopCatcher-MsCHR-RIAD	this study
pET32a-RIDD-ZmCHI	used for protein production and extract preparation of RIDD-ZmCHI	this study

pET32a-ZmPAL-SnoopCatcher	used for protein production and extract preparation of ZmPAL-SnoopCatcher	this study
pET32a-SnoopTag-At4CL4 -CCDIA	used for protein production and extract preparation of SnoopTag-At4CL4 -CCDIA	this study
pET32a-CCDIB-GmCHS-SPyTag003	used for protein production and extract preparation of CCDIB-GmCHS-SPyTag003	this study
pET32a-SpyCatcher003-MsCHR-RIAD	used for protein production and extract preparation of SpyCatcher003-MsCHR-RIAD	this study
pET32a- SPyTag003-At4CL4-SnoopTag	used for protein production and extract preparation of SPyTag003-At4CL4-SnoopTag	this study
pET32a-SnoopCatcher-GmCHS-CCDIA	used for protein production and extract preparation of SnoopCatcher-GmCHS-CCDIA	this study
pET32a-CCDIB-MsCHR-RIAD	used for protein production and extract preparation of CCDIB-MsCHR-RIAD	this study
pET32a-CCDIB-GmCHS-RIAD	used for protein production and extract preparation of CCDIB-GmCHS-RIAD	this study
pET32a-RIDD-MsCHR-SnoopTag	used for protein production and extract preparation of RIDD-MsCHR-SnoopTag	this study
pET32a-SnoopCatcher-ZmCHI	used for protein production and extract preparation of SnoopCatcher-ZmCHI	this study
pET32a-γPFD-SpyCatcher-SpyCatcher003-CCDIA-SnoopCatcher-RIAD	used for protein production and extract preparation of γPFD-SpyCatcher--SpyCatcher003-CCDIA-SnoopCatcher-RIAD	this study
pET32a-SpyTag-ZmPAL	used for protein production and extract preparation of SpyTag-ZmPAL	this study
pET32a-SpyTag003-At4CL4	used for protein production and extract preparation of SpyTag003-At4CL4	this study

pET32a-CCDIB-GmCHS	used for protein production and extract preparation of CCDIB-GmCHS	this study
pET32a-SnoopTag-MsCHR	used for protein production and extract preparation of SnoopTag-MsCHR	this study
**Strains**	**Description**	**Source**
E. coli BL21star (DE3)	For the induced expression of proteins from genes in the constructed plasmid	Invitrogen
E. coli DH5α	Used for transformation and amplification during the plasmid construction procedure	Invitrogen

Table S2. Primers and plasmid used in this study

Name	Description	Primer	Sequence (5'-3')a)
PET-32A-Pc4CL2-RBS	Via inverse PCR, a ribosome binding site (RBS) was introduced upstream of the Pc4CL2 gene for subsequent assembly into a five-gene polycistronic operon	Fwd	CCCTTTTCGGAATTAAGGAGGTAATAAATATGGGCGATTGCGTTGCCCC
		Rev	ATCCCCATGGCCTTGTCGTCGTCGTCGGTACCCAGATCTGGGCTG
PET-32A-PhCHSA-RBS	Via inverse PCR, a ribosome binding site (RBS) was introduced upstream of the PhCHSA gene for subsequent assembly into a five-gene polycistronic operon	Fwd	CCTTTTCGGAATTAAGGAGGTAATAAATATGGTGACCGTGGAAGAATATC
		Rev	AATTCGGATCCCCATGGCCTTGTCGTCGTCGTCGGTACCCAGATCTGGGCTG
PET-32A-MsCHI-RBS	Via inverse PCR, a ribosome binding site (RBS) was introduced upstream of the MsCHI gene for subsequent assembly into a five-gene polycistronic operon	Fwd	ACCTTTTCGGAATTAAGGAGGTAATAAATATGGCCGCAAGTATTACCGC
		Rev	CGACGAATTCGGATCCCCATGGCCTTGTCGTCGTCGTCGGTACCCAG
PET-32A-MsCHR-RBS	Via inverse PCR, a ribosome binding site (RBS) was introduced upstream of the MsCHR gene for subsequent assembly into a five-gene polycistronic operon	Fwd	TTCTTTTCGGAATTAAGGAGGTAATAAATATGGGTAGTGTTGAAATTCC
		Rev	GCTTGTCGACGAATTCGGATCCCCATGGCCTTGTCGTCGTCGTCGGTAC
pET32a-SUP35-SpyCatcher	The pET-32a plasmid backbone was amplified by PCR to add homology arms for Gibson Assembly.	Fwd	TGTAAATGGTTAAGGATCCCACCACCACCACCACCACTGAGATCC
		Rev	ACATCATGGCCTTGTCGTCGTCGTCGGTACCCAGATCTGGGCTG

	Homology arms were added to the SUP35-SpyCatcher fragment by PCR, facilitating its seamless cloning into the pET32a vector via Gibson Assembly.	Fwd	ACGACAAGGCCATGATGTCAGATTCAAATCAAGGTAATAATC
		Rev	GATCCTTAACCATTTACAGTAACCTGACCTTGTTCATTAACTG
	The pET-32a plasmid backbone was amplified by PCR to add homology arms for Gibson Assembly.	Fwd	ACTGTAAATGGTTAACTGATATCGGATCCGCGGCCGCAC
		Rev	TCCATGGAGCCTTGTCGTCGTCGTCGGTACCCAGATC
pET32a-SCVE-SpyCatcher	Homology arms were added to the SCVE-SpyCatcher fragment by PCR, facilitating its seamless cloning into the pET32a vector via Gibson Assembly.	Fwd	CGACAAGGCTCCATGGATGTATAGTTTTGTAAGTGAAG
		Rev	CAGTTAACCATTTACAGTAACCTGACCTTGTTCATTAAC
	The pET-32a plasmid backbone was amplified by PCR to add homology arms for Gibson Assembly.	Fwd	ATATCGGATCCGCGGCCGCACTCGAGCACCACCACCACCACCAC
		Rev	GCATAGCCATGGCCTTGTCGTCGTCGTCGGTACCCAGATCTG
pET32a-P9-SpyCatcher	Homology arms were added to the P9-SpyCatcher fragment by PCR, facilitating its seamless cloning into the pET32a vector via Gibson Assembly.	Fwd	ACAAGGCCATGGCTATGCCATTTCCTTTAGTAAAACAAGATCC
		Rev	CGGCCGCGGATCCGATATCTTAACCATTTACAGTAACCTGAC
pET32a-P22-SpyCatcher	The pET-32a plasmid backbone was amplified by PCR to add homology arms for Gibson Assembly.	Fwd	TGTAAATGGTTAAGATATCGGATCCGCGGCCGCACTCGAGCAC
		Rev	GGCCTTGTCGTCGTCGTCGGTACCCAGATCTGGGCTGTCCATG

	Homology arms were added to the P22-SpyCatcher fragment by PCR, facilitating its seamless cloning into the pET32a vector via Gibson Assembly.	Fwd	ACGACGACGACAAGGCCATGGCTATGGCTTTGAATGAAGGTC
		Rev	ATATCTTAACCATTTACAGTAACCTGACCTTGTTCATTAACTG
	The pET-32a plasmid backbone was amplified by PCR to add homology arms for Gibson Assembly.	Fwd	AGGCCATGGCTATGGCATCTAATTTTACCCAGTTCGTACTGG
		Rev	CGGATCCGATATCAGTTAACCATTTACAGTAACCTGACCTTG
pET32a-MS2-SpyCatcher	Homology arms were added to the MS2-SpyCatcher fragment by PCR, facilitating its seamless cloning into the pET32a vector via Gibson Assembly.	Fwd	TAACTGATATCGGATCCGCGGCCGCACTCGAGCACCACCACCAC
		Rev	ATGCCATAGCCATGGCCTTGTCGTCGTCGTCGGTACCCAGATC
	The pET-32a plasmid backbone was amplified by PCR to add homology arms for Gibson Assembly.	Fwd	GATATCGGATCCGAATTCGAGCTCCGTCGACAAGCTTGC
		Rev	TAGCCATGGCCTTGTCGTCGTCGTCGGTACCCAGATC
pET32a-HBCag-SpyCatcher	Homology arms were added to the HBCag-SpyCatcher fragment by PCR, facilitating its seamless cloning into the pET32a vector via Gibson Assembly.	Fwd	GACAAGGCCATGGCTATGGATATTGATCCTTATAAAG
		Rev	GAATTCGGATCCGATATCTTAACCATTTACAGTAACCTG
pET32a-EutM-SpyCatcher	The pET-32a plasmid backbone was amplified by PCR to add homology arms for Gibson Assembly.	Fwd	GTAAATGGTTAAGATATCGGATCCGCGGCCGCACTCGAGC
		Rev	CTTCCATAGCCATGGCCTTGTCGTCGTCGTCGGTACCCAG

	Homology arms were added to the EutM-SpyCatcher fragment by PCR, facilitating its seamless cloning into the pET32a vector via Gibson Assembly.	Fwd	GGCCATGGCTATGGAAGCATTAGGTATGATTGAAACTC
		Rev	GATATCTTAACCATTTACAGTAACCTGACCTTGTTCATTAAC
	The pET-32a plasmid backbone was amplified by PCR to add homology arms for Gibson Assembly.	Fwd	CTCGAGCACCACCACCACCACCACTGAGATCCGGCTGC
		Rev	AGCCATGGCCTTGTCGTCGTCGTCGGTACCCAGATCTG
pET32a-γPFD-SpyCatcher	Homology arms were added to the γPFD-SpyCatcher fragment by PCR, facilitating its seamless cloning into the pET32a vector via Gibson Assembly.	Fwd	GACGACAAGGCCATGGCTATGGTAAATGAAGTTATTG
		Rev	GGTGGTGGTGCTCGAGTTAACCATTTACAGTAACCTG
	The pET32a-ZmPAL plasmid backbone was amplified by PCR to add homology arms for Gibson Assembly.	Fwd	CGCTCATACTTAAGGATCCCACCACCACCACCACCACTGAGATCC
		Rev	GGTAACACCTGAGCCACCACTACCTTTAATATTAATCGGCAGCGG
pET32aZmPAL-SpyCatcher003	Homology arms were added to the SpyCatcher003 fragment by PCR, facilitating its seamless cloning into the pET32a-ZmPAL vector via Gibson Assembly.	Fwd	GTGGTGGCTCAGGTGTTACCACTCTGTCTGGTCTGTCTGGTGAAC
		Rev	GGGATCCTTAAGTATGAGCGTCACCTTCAGTCGCTTCACCGTCAA
pET32a-SPyTag003-	The SPyTag003 fragment was inserted into the pET-	Fwd	ATACAAACGTTACAAAGGATCTGGTGGTAGTGGTATGCGTCGTATCGCAGCTGGTATCC

At4CL4-CCDIA	32a-At4CL4 backbone plasmid via PCR	Rev	GCGTCAACCATCACGATGTGTGGAACACCACGCATGGATCCGATATCAGCCATGGCCTT
	The pET-32a-SPyTag003-At4CL4 plasmid backbone was amplified by PCR to add homology arms for Gibson Assembly.	Fwd	TAAGAATTCGAGCTCCGTCGACAAGCTTGCGGCCGCACTCGAGCACCACC
		Rev	GACCACCTGAGCCACCACTACCCTTAGAGCACATGGTTTCCAGCTTTGCACG
	Homology arms were added to the CCDIA fragment by PCR, facilitating its seamless cloning into the pET-32a-SPyTag003-At4CL4 vector via Gibson Assembly.	Fwd	AGGGTAGTGGTGGCTCAGGTGGTCTGGAACAAGAAATCGCTGCGCTGGAG
		Rev	CTTGTCGACGGAGCTCGAATTCTTAACCACCACGTTCCAGCGCCGCGATTTCCCA
pET32a-CCDIB--GmCHS-SnoopTag	The pET-32a-GmCHS plasmid backbone was amplified by PCR to add homology arms for Gibson Assembly.	Fwd	GGTAGTGGTGGTAGTGGTATGGTTAGCGTTGCCGAAATTCGTC
		Rev	CCAGGAATTCGGATCCCCATGGCCTTGTCGTCGTCGTCGGTACCC
	Homology arms were added to the CCDIB fragment by PCR, facilitating its seamless cloning into the pET-32a-GmCHS vector via Gibson Assembly.	Fwd	CATGGGGATCCGAATTCCTGGTAAAATCGCGGCTCTGAAACAGAA
		Rev	ACCATACCACTACCACCACTACCACCACCCTGTTTCAGCGC
	The SnoopTag fragment was inserted into the pET-32a-CCDIB-GmCHS backbone plasmid via PCR	Fwd	TGATATTGAATTTATTAAAGTTAATAAATAAAAGCTTCTCGAGCACCACCACCAC
		Rev	CCTAATTTCATACCTGAGCCACCACTACCAATGGCAACGCTATGCAGCACCACGG
	Homology arms were added to the SnoopCatcher	Fwd	CGTCGACAAGCTTCTATGAAACCATTACGTGGTGCTGTATTTAGT

	fragment by PCR, facilitating its seamless cloning into the pET-32a-MsCHR vector via Gibson Assembly.	Rev	ACCCATGCCTGATCCTCCAGAGCCTTTTGGTGGAATTGGTTCATTAGTGATATAATG
	The pET-32a-MsCHR plasmid backbone was amplified by PCR to add homology arms for Gibson Assembly.	Fwd	ACCAAAAGGCTCTGGAGGATCAGGCATGGGTAGTGTTGAAATTCCGACCAAAGTGCTG
		Rev	GGTTTCATAGAAGCTTGTCGACGGAGCTCGAATTCGGATCCGATATC
pET32a-SnoopCatcher-MsCHR-RIAD	Homology arms were added to the RIAD fragment by PCR, facilitating its seamless cloning into the pET-32a-SnoopCatcher-MsCHR vector via Gibson Assembly.	Fwd	ATGATGATGGCAGTGGTGGATCAGGGATGtTGGAACAATACGCTAACCAATTGGC
		Rev	GTGCTCGAGTGCGGCCGCTTAACAACCTTCAGTAGCTTCCTTGATGATTTGATCA
	The pET-32a-SnoopCatcher-MsCHR plasmid backbone was amplified by PCR to add homology arms for Gibson Assembly.	Fwd	GTTAAGCGGCCGCACTCGAGCACCACCACCACCACCACTGAGATCCGGCTGC
		Rev	TGATCCACCACTGCCATCATCATACAGATCATTCAGGCCCGGTTTGGTCGGACCC
pET32a-RIDD-ZmCHI	Homology arms were added to the RIDD fragment by PCR, facilitating its seamless cloning into the pET-32a-ZmCHI vector via Gibson Assembly.	Fwd	TCCGTCGACCTATGTCCTTGAGAGAATGTGAATTGTACGTTCAA
		Rev	CCTGAGCCtCCACTACCCTTAGCTTCTTCCTTTTCCAATCTTTC
	The pET-32a-ZmCHI plasmid backbone was amplified by PCR to add homology arms for Gibson Assembly.	Fwd	AGGGTAGTGGaGGCTCAGGTATGGCATGTCGCCGCTGGTGGAGCAC
		Rev	CTCTCAAGGACATAGGTCGACGGAGCTCGAATTCGGATCCGATA

pET32a-ZmPAL -SnoopCatcher	Homology arms were added to the SnoopCatcher fragment by PCR, facilitating its seamless cloning into the pET-32a-ZmPAL vector via Gibson Assembly.	Fwd	TAGTGGTGGCTCAGGTATGAAACCATTACGTGGTGCTGTA
		Rev	CGGATCCTTATTTGGTGGAATTGGTTCATTAGTGATATAAT
	The pET-32a-ZmPAL plasmid backbone was amplified by PCR to add homology arms for Gibson Assembly.	Fwd	TCCACCAAAATAAGGATCCGAATTCGTCGACAAGCTTCTCG
		Rev	ACCTGAGCCACCACTACCTTTAATATTAATCGGCAGCGGTT
pET32a-SnoopTag-At4CL4 -CCDIA	Homology arms were added to the CCDIA fragment by PCR, facilitating its seamless cloning into the pET-32a-At4CL4 vector via Gibson Assembly.	Fwd	AGGGTAGTGGTGGCTCAGGTGGTCTGGAACAAGAAATCGCTGCGCTGGAG
		Rev	CCGGATCTCAGTGGTGGTGGTGGTGGTGGAATTCACCACCACGTTCCAG
	The pET-32a-At4CL4 plasmid backbone was amplified by PCR to add homology arms for Gibson Assembly.	Fwd	ACCACCACCACTGAGATCCGGCTGCTAACAAAGCCCGAAAGGAAGCTGAG
		Rev	GACCACCTGAGCCACCACTACCCTTAGAGCACATGGTTTCCAGCTTTGCACG
	Homology arms were added to the SnoopTag fragment by PCR, facilitating its seamless cloning into the pET-At4CL4-CCDIA vector via Gibson Assembly.	Fwd	AGTTAATAAAGGTAGTGGTGGCTCAGGTATGCGTCGTATCGCAGCTGGTATC
		Rev	TTAATAAATTCAATATCACCTAATTTCATAGGGATCCCCATGGCCTTGTCG
pET32a-CCDIB-	The SpyTag003 fragment was inserted into the	Fwd	TCGTGATGGTTGACGCATACAAACGTTACAAATAAGTCGACAAGCTTCTCGAGCACCA

GmCHS-SPyTag003	pET32a-CCDIB-GmCHS backbone plasmid via PCR	Rev	TGTGTGGAACACCACGACCACTACCACCAGATCCAATGGCAACGCTATGCAGCACCAC
	Homology arms were added to the SpyCatcher003 fragment by PCR, facilitating its seamless cloning into the pET32a-MsCHR vector via Gibson Assembly.	Fwd	AGCTTCTGTTACCACTCTGTCTGGTCTGTCTGGTGAACA
		Rev	CCTCCAGAGCCAGTATGAGCGTCACCTTCAGTCGCTTCACC
pET32a-SPCatcher003-MsCHR-RIAD	The pET-32a-MsCHR plasmid backbone was amplified by PCR to add homology arms for Gibson Assembly.	Fwd	ATACTGGCTCTGGAGGATCAGGCATGGGTAGTGTTGAAATT
		Rev	AGTGGTAACAGAAGCTTGATATCAGCCATGGCCTTGTCGTC
	Homology arms were added to the RIAD fragment by PCR, facilitating its seamless cloning into the pET32a-SpyCatcher003-MsCHR vector via Gibson Assembly.	Fwd	GGTGGATCAGGGATGTTGGAACAATACGCTAACCAATTGGCTGATCAAATC
		Rev	GTGGTGGTGCTCGAGTGCGGCCGCTTAACAACCTTCAGTAGCTTCCTTGAT
	The pET-32a-SpyCatcher003-MsCHR plasmid backbone was amplified by PCR to add homology arms for Gibson Assembly.	Fwd	GCACTCGAGCACCACCACCACCACCACTGAGATCCGGCTGCTAACAAAG
		Rev	CAACATCCCTGATCCACCACTGCCATCATCATACAGATCATTCAGGCCCGG
pET32a-SPyTag 003-At4CL4-SnooPTag	The SnooPTag fragment was inserted into the pET32a-SPyTag003-At4CL4 backbone plasmid via PCR	Fwd	GGTGATATTGAATTTATTAAAGTTAATAAATAAGAATTCGAGCTCCGTCGACAAGCT
		Rev	TAATTTCATACCTGAGCCACCACTACCCTTAGAGCACATGGTTTCCAGCTTTG
pET32a-SnooPCatcher--	Homology arms were added to the SnooPCatcher	Fwd	GGGGATCCGAATTCCTATGAAACCATTACGTGGTGC

GmCHS-CCDIA	fragment by PCR, facilitating its seamless cloning into the pET32a-GmCHS vector via Gibson Assembly.	Rev	CCATACCACTACCACCACTACCTTTTGGTGGAATTGG
	The pET-32a-GmCHS plasmid backbone was amplified by PCR to add homology arms for Gibson Assembly.	Fwd	GGTGGTAGTGGTATGGTTAGCGTTGCCGAAATTCG
		Rev	ATAGGAATTCGGATCCCCATGGCCTTGTCGTCGTCGTC
	Homology arms were added to the CCDIA fragment by PCR, facilitating its seamless cloning into the pET32a-SnooPCatcher-GmCHS vector via Gibson Assembly.	Fwd	GCCATTGGTAGTGGTGGCTCAGGTGGTCTGGAACAAGAAATC
		Rev	GGTGGTGCTCGAGAAGCTTTTAACCACCACGTTCCAGCGC
	The pET-32a-SnooPCatcher-GmCHS plasmid backbone was amplified by PCR to add homology arms for Gibson Assembly.	Fwd	AGCTTCTCGAGCACCACCACCACCACCACTGAGATCCGGC
		Rev	GCCACCACTACCAATGGCAACGCTATGCAGCACCACGGTTTC
pET32a-CCDIB-MsCHR-RIAD	Homology arms were added to the CCDIB fragment by PCR, facilitating its seamless cloning into the pET32a-MsCHR-RIAD vector via Gibson Assembly.	Fwd	TTCGTCGACAAGCTTCTGGTAAAATCGCGGCTCTG
		Rev	TGAGCCGCCAGAGCCACCACCCTGTTTCAGCGCC
	The pET-32a-MsCHR-RIAD plasmid backbone was amplified by PCR to add homology arms for Gibson Assembly.	Fwd	GGCTCTGGCGGCTCAGGCATGGGTAGTGTTGAAATTCC
		Rev	AGAAGCTTGTCGACGAATTCGGATCCCCATGGCCTTG

pET32a-CCDIB-GmCHS-RIAD	Homology arms were added to the RIAD fragment by PCR, facilitating its seamless cloning into the pET32a-CCDIB-GmCHS vector via Gibson Assembly.	Fwd	TAGTGGTGGCTCAGGTATGTTGGAACAATACGCTAAC
		Rev	GAAGCTTTTAACAACCTTCAGTAGCTTCCTTG
	The pET32a-CCDIB-GmCHS plasmid backbone was amplified by PCR to add homology arms for Gibson Assembly.	Fwd	GGTTGTTAAAAGCTTCTCGAGCACCACCACCAC
		Rev	TACCTGAGCCACCACTACCAATGGCAACGCTATGCAG
pET32a-RIDD-MsCHR-SnooPTag	Homology arms were added to the RIDD fragment by PCR, facilitating its seamless cloning into the pET32a-MsCHR vector via Gibson Assembly.	Fwd	GTCGACAAGCTTCTTCCTTGAGAGAATGTGAATTG
		Rev	TGCCTGAGCCGCCAGAGCCCTTAGCTTCTTCCTTTTCC
	The pET-32a-MsCHR plasmid backbone was amplified by PCR to add homology arms for Gibson Assembly.	Fwd	CTCTGGCGGCTCAGGCATGGGTAGTGTTGAAATTCCG
		Rev	GAAGAAGCTTGTCGACGAATTCGGATCCCCATGGC
	The SnooPTag fragment was inserted into the pET32a-RIDD-MsCHR backbone plasmid via PCR	Fwd	GTGATATTGAATTTATTAAAGTTAATAAATAACTCGAGCACCACCACCACCAC
		Rev	CTAATTTCATACCTGAGCCACCACTACCATCATCATACAGATCATTCAGGCCC
pET32a-SnooPCatcher-ZmCHI	Homology arms were added to the SnooPCatcher fragment by PCR, facilitating its seamless cloning into the pET32a-ZmCHI vector via Gibson Assembly.	Fwd	ATTCGTCGACCTATGATGAAACCATTACGTGGTGC
		Rev	GCCACCACTACCTTTTGGTGGAATTGGTTCATTAG

	The pET-32a-ZmCHI plasmid backbone was amplified by PCR to add homology arms for Gibson Assembly.	Fwd	AAAGGTAGTGGTGGCTCAGGTATGGCATGTCGCCGCTG
		Rev	CATAGGTCGACGAATTCGGATCCCCATGGCCTTGTC
	The pET-32a-γPFD-SpyCatcher plasmid backbone was amplified by PCR to add homology arms for Gibson Assembly.	Fwd	CTCGAGCACCACCACCACCACCACTGAGATCCGGCTGC
		Rev	CACCTGAGCCACCACTACCACCATTTACAGTAACCTGAC
pET32a-γPFD-SpyCatcher-SpyCatcher003--CCDIA-SnoopCatcher-RIAD	The fusion fragment SpyCatcher003-CCDIA-SnoopCatcher-RIAD was obtained by PCR amplification.	Fwd	AGCGACTGAAGGTGACGCTCATACTGGTAGTGGTGGTAGTGGTGGTCTGGAAC
		Rev	AGCGTTCTCTTTCTCCAGCGCAGCGATTTCTTGTTCCAGACCACCACTACCACCACTAC
		Fwd	CGCTGCGCTGGAGAAAGAGAACGCTGCTCTGGAATGGGAAATCGCGGCGCTGGAACGTG
		Rev	CCTAATTTCATGCCTGATCCTCCAGAGCCACCACCACGTTCCAGCGCCGCGATTTCCC
		Fwd	CTGGAGGATCAGGCATGAAATTAGGTGATATTGAATTTATTAAAGTTAATAAAGGCAG
		Rev	TTAGCGTATTGTTCCAACATCCCTGATCCACCACTGCCTTTATTAACTTTAATAAATTC
		Fwd	CAGGGATGTTGGAACAATACGCTAACCAATTGGCTGATCAAATCATCAAGGAAGCTAC
		Rev	TGGTGGTGGTGGTGCTCGAGTTAACAACCTTCAGTAGCTTCCTTGATGATTTGATCAG

	Homology arms were added to the SpyCatcher003-CCDIA-SnoopCatcher-RIAD fragment by PCR, facilitating its seamless cloning into the pET32a-γPFD-SpyCatcher vector via Gibson Assembly.	Fwd	GTAGTGGTGGCTCAGGTGTTACCACTCTGTCTGGTCTG
		Rev	GGTGGTGGTGCTCGAGTTAACAACCTTCAGTAGCTTCC
pET32a-SpyTag-ZmPAL	The SpyTag fragment was inserted into the pET32a-ZmPAL backbone plasmid via PCR	Fwd	AAACCTACTAAAGGATCTGGTGGTAGTGGTATGGCAGGTAATGGTGCCATTGTTG
		Rev	ATATGCATCAACCATAACGATGTGTGCCATAGCCATGGCCTTGTCGTCGTCGTCG
pET32a-SpyTag-At4CL4	The SpyTag fragment was inserted into the pET32a-At4CL4 backbone plasmid via PCR	Fwd	AAACCTACTAAAGGATCTGGTGGTAGTGGTATGCGTCGTATCGCAGCTGGTATCC
		Rev	ATATGCATCAACCATAACGATGTGTGCCATAGGGATCCCCATGGCCTTGTCGTCG
pET32a-SpyTag-GmCHS	The SpyTag fragment was inserted into the pET32a-GmCHS backbone plasmid via PCR	Fwd	AAACCTACTAAAGGATCTGGTGGTAGTGGTATGGTTAGCGTTGCCGAAATTCGTC
		Rev	ATATGCATCAACCATAACGATGTGTGCCATAGGAATTCGGATCCCCATGGCCTTGTCG
pET32a-SpyTag-MsCHR	The SpyTag fragment was inserted into the pET32a-MsCHR backbone plasmid via PCR	Fwd	TAAACCTACTAAAGGATCTGGTGGTAGTGGTATGGGTAGTGTTGAAATTCCGACC
		Rev	TATGCATCAACCATAACGATGTGTGCCATAGAAGCTTGTCGACGAATTCGGATCCC
pET32a-SpyTag-ZmCHI	The SpyTag fragment was inserted into the pET32a-ZmCHI backbone plasmid via PCR	Fwd	ATATAAACCTACTAAAGGATCTGGTGGTAGTGGTATGGCATGTCGCCGCTGGTGGAG
		Rev	GCATCAACCATAACGATGTGTGCCATAGGTCGACGAATTCGGATCCCCATGGCCTTG

pET32a-ZmPAL-SpyTag	The SpyTag fragment was inserted into the pET32a-ZmPAL backbone plasmid via PCR	Fwd	TGGTTGATGCATATAAACCTACTAAACACCACCACCACCACCACTGAGATCCGGCTGC
		Rev	TAACGATGTGTGCCATACCACTACCACCAGATCCTTTAATATTAATCGGCAGCGGTTTG
pET32a-At4CL4-SpyTag	The SpyTag fragment was inserted into the pET32a-At4CL4 backbone plasmid via PCR	Fwd	GGTTGATGCATATAAACCTACTAAACACCACCACCACCACCACTGAGATCCGGCTG
		Rev	ATAACGATGTGTGCCATACCACTACCACCAGATCCCTTAGAGCACATGGTTTCCAGC
pET32a-GmCHS-SpyTag	The SpyTag fragment was inserted into the pET32a-GmCHS backbone plasmid via PCR	Fwd	CGTTATGGTTGATGCATATAAACCTACTAAACACCACCACCACCACCACTGAGATCCGG
		Rev	ATGTGTGCCATACCACTACCACCAGATCCAATGGCAACGCTATGCAGCACCACGGTTTC
pET32a-MsCHR-SpyTag	The SpyTag fragment was inserted into the pET32a-MsCHR backbone plasmid via PCR	Fwd	ATGGTTGATGCATATAAACCTACTAAACTCGAGCACCACCACCACCACCACTGAGATC
		Rev	AACGATGTGTGCCATACCACTACCACCAGATCCATCATCATACAGATCATTCAGGCCC
pET32a-ZmCHI-SpyTag	The SpyTag fragment was inserted into the pET32a-ZmCHI backbone plasmid via PCR	Fwd	TTATGGTTGATGCATATAAACCTACTAAACACCACCACCACCACCACTGAGATCCGGC
		Rev	CGATGTGTGCCATACCACTACCACCAGATCCTGCGGTAATGCTAACCGGTTCTGCCTG

Table S3. 9-Fold Cross-Validation Results

Round	Model	Mean_R2	Std_R2	Mean_RMSE	Std_RMSE
3	Ensemble_Stack	0.902584	0.060753	13.171514	5.680165
3	Ensemble_NNLS	0.898513	0.06789	13.248208	6.367381
3	SVR	0.89602	0.072709	13.331667	6.697996
3	Ensemble_Avg	0.891841	0.0615	13.836935	5.829
3	XGBoost	0.891713	0.047193	13.991548	4.546927
3	RandomForest	0.880941	0.043232	14.778039	4.380765
3	CatBoost	0.866528	0.078049	15.381712	6.597276
3	LightGBM	0.860501	0.055769	15.949434	4.855415
3	GPR	0.843307	0.088587	16.754631	7.013728
3	MLP	0.794193	0.13225	18.704939	7.706373
2	XGBoost	0.825478	0.079378	13.071962	4.17195
2	LightGBM	0.797427	0.145378	13.218375	3.945705
2	RandomForest	0.788947	0.131002	13.887099	3.816775
2	CatBoost	0.743977	0.134179	15.633411	5.178692
2	GPR	0.666905	0.25896	16.379103	6.63182
2	MLP	0.622162	0.213	18.970075	6.94228
2	SVR	0.253183	0.223794	29.467647	12.40902
1	GPR	0.14098	1.426159	12.766733	6.484949
1	CatBoost	0.054449	1.777039	12.916636	5.359265
1	XGBoost	0.018963	1.653649	13.867492	5.379248
1	RandomForest	-0.111244	2.32001	12.812231	5.003337
1	SVR	-0.112344	0.205296	22.617872	8.052081
1	MLP	-0.169636	1.457679	16.545823	7.195678
1	LightGBM	-1.108038	5.362024	13.315991	6.480751
